# Simple analytical model reveals the functional role of embodied sensorimotor interaction in hexapod gaits

**DOI:** 10.1371/journal.pone.0192469

**Published:** 2018-02-28

**Authors:** Yuichi Ambe, Shinya Aoi, Timo Nachstedt, Poramate Manoonpong, Florentin Wörgötter, Fumitoshi Matsuno

**Affiliations:** 1 Department of Applied Information Sciences, Graduate School of Information Sciences, Tohoku University, Sendai, Japan; 2 Department of Aeronautics and Astronautics, Graduate School of Engineering, Kyoto University, Kyoto, Japan; 3 Bernstein Center for Computational Neuroscience, Third Institute of Physics, Georg-August-Universität Göttingen, Göttingen, Germany; 4 Embodied AI and Neurorobotics Lab, Centre for Biorobotics, The Mærsk Mc-Kinney Møller Institute, University of Southern Denmark, Odense M, Denmark; 5 Bio-inspired Robotics and Neural Engineering Lab, School of Information Science and Technology, Vidyasirimedhi Institute of Science and Technology, Rayong, Thailand; 6 Department of Mechanical Engineering and Science, Graduate School of Engineering, Kyoto University, Kyoto, Japan; Georgia State University, UNITED STATES

## Abstract

Insects have various gaits with specific characteristics and can change their gaits smoothly in accordance with their speed. These gaits emerge from the embodied sensorimotor interactions that occur between the insect’s neural control and body dynamic systems through sensory feedback. Sensory feedback plays a critical role in coordinated movements such as locomotion, particularly in stick insects. While many previously developed insect models can generate different insect gaits, the functional role of embodied sensorimotor interactions in the interlimb coordination of insects remains unclear because of their complexity. In this study, we propose a simple physical model that is amenable to mathematical analysis to explain the functional role of these interactions clearly. We focus on a foot contact sensory feedback called phase resetting, which regulates leg retraction timing based on touchdown information. First, we used a hexapod robot to determine whether the distributed decoupled oscillators used for legs with the sensory feedback generate insect-like gaits through embodied sensorimotor interactions. The robot generated two different gaits and one had similar characteristics to insect gaits. Next, we proposed the simple model as a minimal model that allowed us to analyze and explain the gait mechanism through the embodied sensorimotor interactions. The simple model consists of a rigid body with massless springs acting as legs, where the legs are controlled using oscillator phases with phase resetting, and the governed equations are reduced such that they can be explained using only the oscillator phases with some approximations. This simplicity leads to analytical solutions for the hexapod gaits via perturbation analysis, despite the complexity of the embodied sensorimotor interactions. This is the first study to provide an analytical model for insect gaits under these interaction conditions. Our results clarified how this specific foot contact sensory feedback contributes to generation of insect-like ipsilateral interlimb coordination during hexapod locomotion.

## Introduction

**Fig 1:**
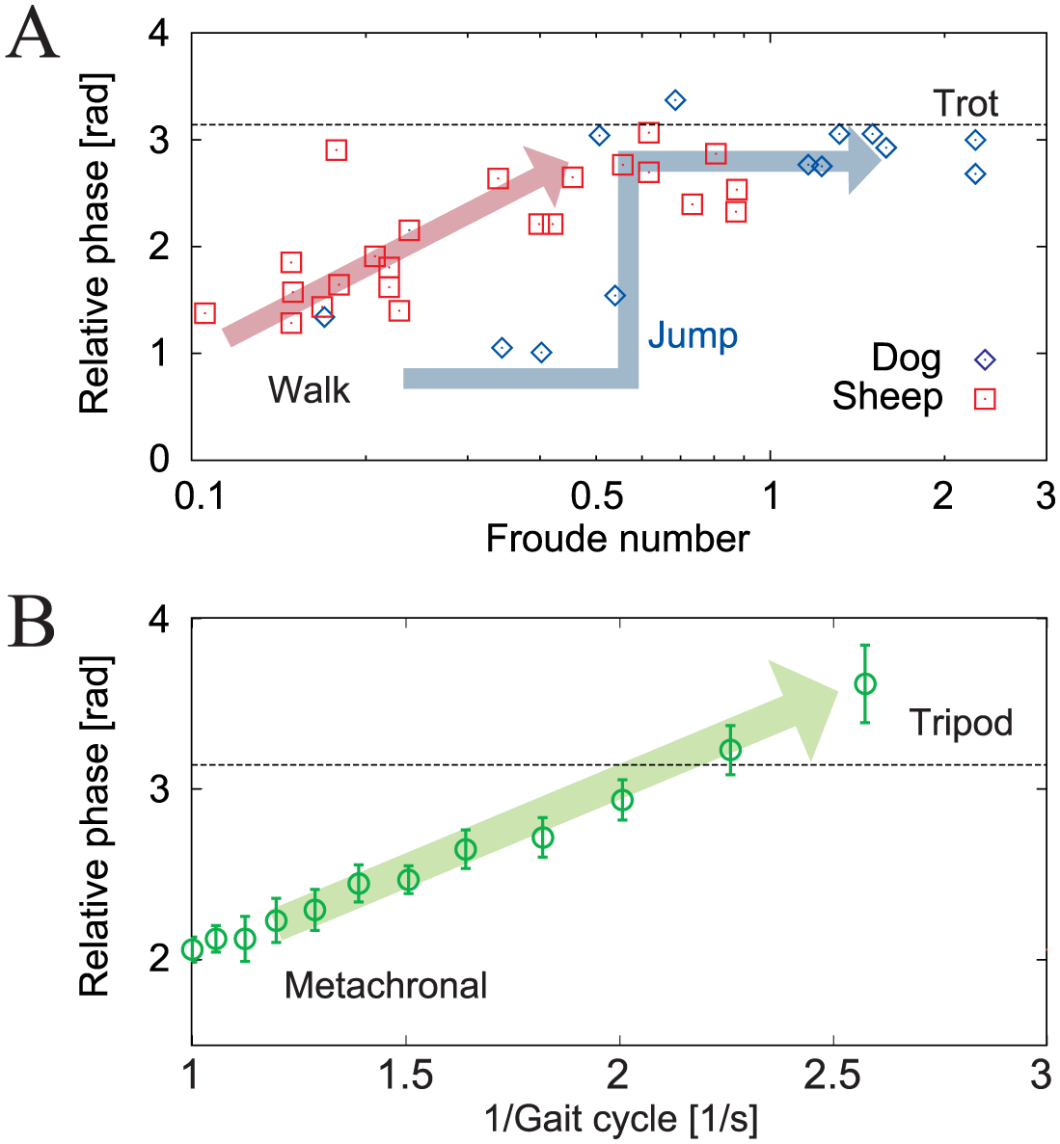
Interlimb phase relationships for locomotion speed. **A**: Ipsilateral relative phases (fore leg–hind leg) for dogs and sheep versus Froude number (where the locomotion speed increases as the Froude number increases) [3]. Dogs change their phase relationship suddenly at a Froude number of approximately 0.5, while sheep change their phase relationship smoothly based on locomotion speed. **B**: Ipsilateral relative phases ((fore leg–hind leg)/2) of stick insects for gait cycle (where the locomotion speed decreases as the gait cycle increases) [4]. Data points and error bars show the average values and the errors of the mean values of the measured results, respectively. Stick insects change their phase relationships smoothly based on locomotion speed in a manner similar to sheep.

Legged animals prefer specific gaits and change these gaits in accordance with their locomotion speeds. For example, quadruped animals use a walking gait at lower speeds but use a trotting gait at higher speeds. These gaits are characterized by the relative phases between the limbs (called interlimb phase relationship) [1, 2]. In the walking gait, the swinging movements of the legs propagate from back to front, while in the trotting gait, the diagonal legs move in phase. During the transition between these gaits, some quadrupeds, such as dogs, change their ipsilateral phase relationships instantly in a manner similar to the human walk–run transition, whereas other quadrupeds, such as sheep, change their phase relationship with a smooth transition depending on their locomotion speed, as shown in Fig 1A [3].

Insects also change their gaits, e.g., between metachronal and tripod gaits, depending on their locomotion speeds, as observed in stick insects [4–7], cockroaches [8–10], and flies [11]. Like quadrupeds, the gaits of insects are also characterized by the relative phases between their limbs. In the metachronal gait, the swinging movements of the legs propagate from posterior to anterior in a manner similar to the quadrupedal walking gait (we call this gait the direct wave gait), while in the tripod gait, the diagonal legs move in phase, like the quadrupedal trotting gait. There is a near-antiphase relationship between the left and right limbs, irrespective of the locomotion speed. While insects can choose gaits in which the swinging movements of the legs propagate from anterior to posterior (we call this gait the retrograde wave gait), as observed in some centipedes [12], they do not use the retrograde wave gait and prefer to use the direct wave gait like quadrupeds. Furthermore, similar to sheep, insects change their ipsilateral relative phases smoothly based on their locomotion speed, as shown in Fig 1B.

Locomotion is a complex process that requires various components with real-time interaction between motor control functions and body dynamics through sensory feedback (embodied sensorimotor interaction) [13]. For example, it is known that stick insects do not generate coordinated motor outputs without sensory feedback [14, 15], which indicates that sensory feedback plays a critical role in shaping these motor patterns. To attempt to understand the locomotion mechanisms of insects, many researchers have developed bio-inspired control models that use sensory feedback. It is important to clarify the functional role of sensory feedback, because sensory feedback has been shown to be an important factor in adaptive and coordinated leg movements in many studies [16–21]. Cruse and colleagues [22–25] identified six rules required to establish interlimb coordination based on behavioral studies, and proposed a bio-inspired controller using an artificial neural network, called Walknet. This network creates various movements for hexapod models and robots, including gait transitions, curve walking and searching behavior, as observed in stick insects. Daun-Gruhn [26] developed an oscillator network model of stick insect walking based on use of central pattern generators (CPGs) for each leg joint along with sensory feedback, which generated the insect like gaits by introducing excitatory and inhibitory synaptic connections among the oscillators for the ipsilateral front to rear legs. Neuromechanical models of the insect were also proposed based on physiological findings to demonstrate adaptive walking using sensory feedback [27–29].

While these models can replicate insect gaits, the functional role of sensory feedback in interlimb coordination is still not fully understood. It is not clear when and how sensory feedback affects the insect gaits, which is an important factor in the design of the robotic controller. This is largely because these models are too complex (i.e., they have multiple sensory feedback channels, neurons, and muscles). In particular, the effects of embodied sensorimotor interactions are too complex to be analyzed using these models. Owaki et al. [30] proposed minimal model to describe the hexapedal interlimb coordination solely by using the local and neighboring leg loading information. However, they investigated them experimentally with the robot. To the best of our knowledge, no study to date has investigated this effect analytically (i.e., by representing the effects of sensory motor interaction with an analytical solution for the system). An analytical understanding of this effect would be helpful in providing a deeper understanding of the gait generation mechanism and for the design of the robotic controller.

In this paper, we designed a minimal control model for hexapod locomotion. A single oscillator is used to control the movement of each leg. While the contralateral oscillators are constrained to be antiphase, there is no connection between the ipsilateral oscillators. The ipsilateral coordination is formed by the local sensory feedback (i.e., foot contact information). The local sensory feedback, in the form of phase resetting, modulates the oscillator rhythm based on local tactile information. We investigated the effects of sensory feedback on hexapod gaits using a hexapod robot and found that the robot generated two gaits through the sensory feedback; one of these gaits had the following major characteristic properties of insect gaits [5].

P1 The swing movement propagates from posterior to anterior (i.e., a direct wave gait).
P2 The ipsilateral leg coordination changes smoothly depending on the locomotion speed (i.e., it changes from a metachronal to a tripod gait as the speed increases).

The other gait satisfied P2 but the swing movement propagated from anterior to posterior (i.e., a retrograde wave gait). Next, we propose a simple physical model that is amenable to mathematical analysis to explain the above gait mechanisms analytically. This simple model consists of a rigid body that uses massless springs as legs; the legs are controlled using oscillator phases with phase resetting and its governing equations are reduced such that they can be explained using only the oscillator phases with some approximations. This simplicity allows us to reach analytical solutions for the hexapod gaits via perturbation analysis, despite the complex nature of the embodied sensorimotor interactions.

The main contribution of this work is the elucidation of the functional role of specific foot contact sensory feedback on hexapod gaits using both the real physical robot and the simple model. In particular, we demonstrated that the direct and retrograde wave gaits were produced through local sensory feedback using touchdown information, and these gaits changed smoothly depending on the locomotion speed. These results suggest that the local sensory feedback contributes to generation of insect-like ipsilateral interlimb coordination. Furthermore, we explained these mechanisms analytically using the simple model, which then allowed us to discuss the differences between the direct and retrograde wave gaits, and the reason for the smooth gait transition. In addition, the simple model shows when and how the sensory feedback affects the gaits. Because the simple model proposed here was able to extract the essence of the gait generation mechanism, this simple model analysis can also be applied to future investigations of other sensory feedback mechanisms and legged locomotion systems.

## Results

### Hexapod robot and its controller with sensory feedback

**Fig 2:**
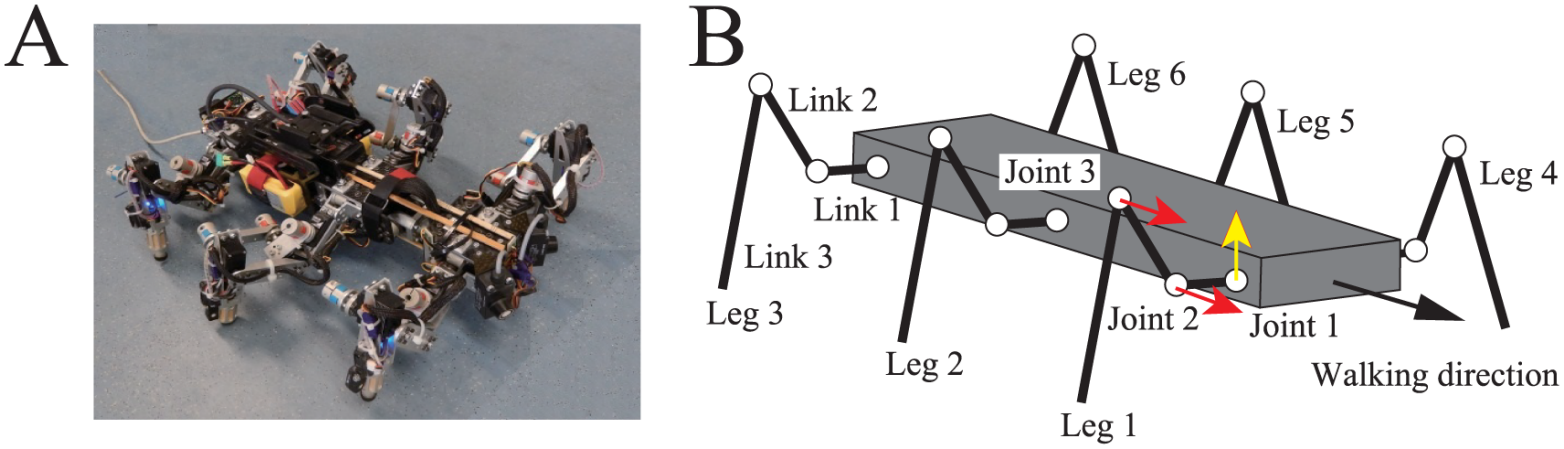
Hexapod robot. **A**: Robot; **B**: Model.

We used a hexapod robot (AMOS II [17]; see Fig 2A) consisting of one body with six legs (Legs 1–6). Fig 2B shows the physical model of the robot that was used for the computer simulations. Joint 1 is a yaw joint that moves the leg from back to front, while joints 2 and 3 are pitch joints that lift the leg up and down. A touch sensor is installed on the tip of each leg.

**Fig 3:**
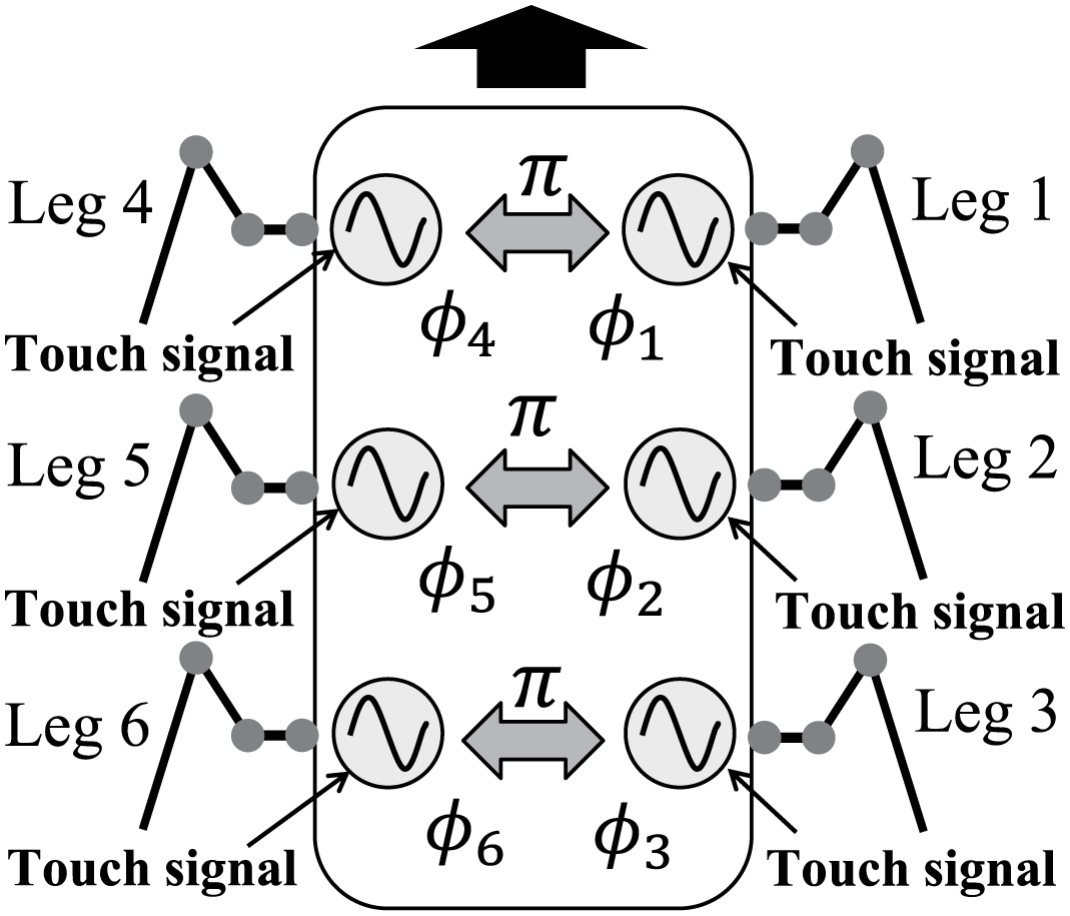
Locomotion control system using phase oscillators. Each oscillator controls the movement of a single leg. Contralateral oscillators are set to have alternate phases. Each oscillator is affected by the touch sensor signal.

**Fig 4:**
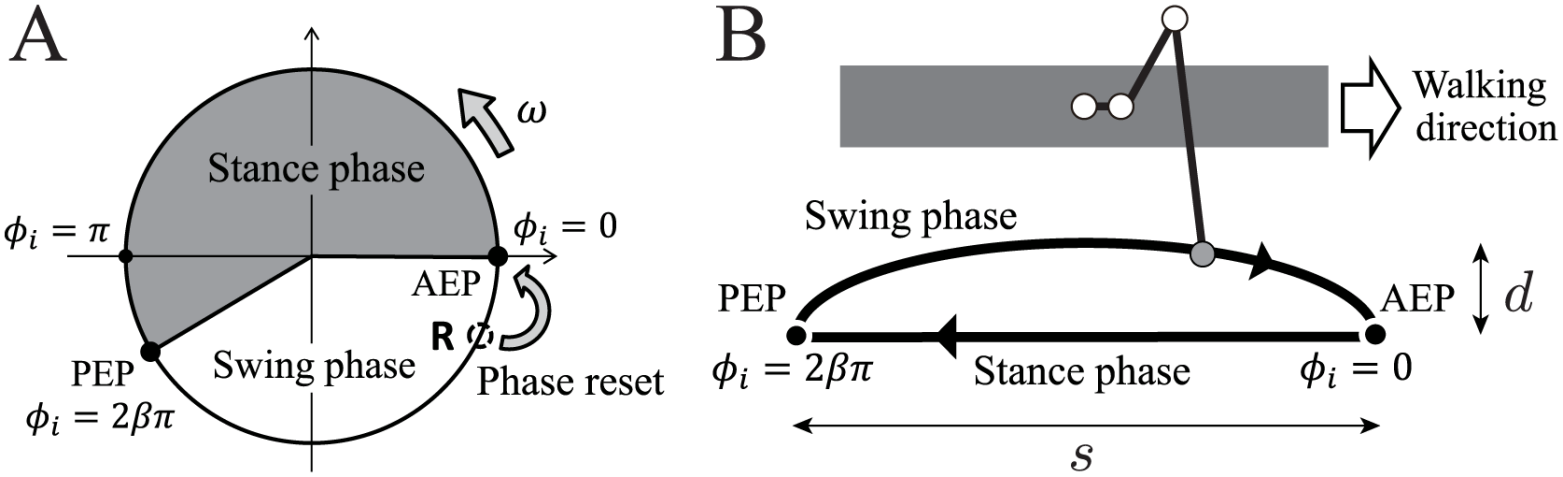
Leg movement based on oscillator phase. **A**: Oscillator phase. **B**: Desired leg movement. AEP and PEP represent the anterior extreme position and the posterior extreme position, respectively.

We developed the control system for this robot using phase oscillators that were inspired by the physiological concepts of CPGs and sensory feedback described in [31–33] (Fig 3). Here, an overview of the system is given. We used six phase oscillators (designated Oscillators 1–6) with phases of *ϕ*_*i*_ (0 ≤ *ϕ*_*i*_ < 2*π*, *i* = 1, …, 6), and designed the trajectory of the tip of each Leg *i* relative to the body using *ϕ*_*i*_ (Fig 4). The trajectory is composed of a line segment with length *s* for the stance phase (0 ≤ *ϕ*_*i*_ < 2*βπ*) and a simple ellipsoid curve with height *d* for the swing phase (2*βπ* ≤ *ϕ*_*i*_ < 2*π*), where *β* is the duty factor (i.e., the ratio between the stance phase and step cycle durations). We set the duration of the swing phase to be *T*_sw_ = const., as is often observed in insects [4, 5]. The walking speed *v* can then be given as *v* = (1 − *β*)*s*/*βT*_sw_. Each joint was controlled using a proportional-derivative (PD) feedback controller to generate the desired joint angle, which was calculated using inverse kinematics.

The phase oscillators have a basic frequency and the phases of these oscillators are modulated based on the interactions among them and the local sensory feedback. In insect gaits, changes in the ipsilateral phase relationships are dependent on speed, while the contralateral phase relationships are almost in antiphase [5]. To ensure that the system is simple, we modeled the interactions such that contralateral oscillators remain in antiphase. However, there are no direct relationships among the ipsilateral oscillators.

Physiological evidence has shown that detection of an increasing load on a leg promotes the retraction of that leg [34, 35], and there are also some interneurons that cause a reset of the rhythmicity in motoneuron activities [36]. Based on these findings, we incorporated the phase resetting mechanism with foot contact signal as the local sensory feedback mechanism [31–33]. More specifically, when Leg *i* touches the ground during the swing phase (2*βπ* ≤ *ϕ*_*i*_ < 2*π*) as indicated by point R in Fig 4A, the phase *ϕ*_*i*_ is reset to zero (see the Materials and Methods section).

Because the leg movements of our robot are determined by these oscillation phases, the relative phases between the oscillators (*ψ*_1_(= *ϕ*_2_ − *ϕ*_1_) and *ψ*_2_(= *ϕ*_3_ − *ϕ*_2_)) thus explain the gait, which is produced by interactions among the oscillators and the sensory feedback. In this study, we varied the locomotion speed *v* using the duty factor *β* to determine whether our robot produced gaits that satisfy insect gait properties P1 and P2 through the embodied sensorimotor interactions using computer simulations and robot experiments; however, these properties were neither predesigned nor predetermined.

### Simulation results

We performed computer simulations using the robot model (Fig 2B) and various locomotion speeds by changing *β* from 0.5 to 0.65 in even steps (where the oscillator frequency changed from 0.05 to 0.0035 Hz, and the locomotion speed changed from 0.015 to 0.008 body lengths per second). At each locomotion speed, stable gaits were found by changing the various initial values of the relative phases (*ψ*_1_, *ψ*_2_).

**Fig 5:**
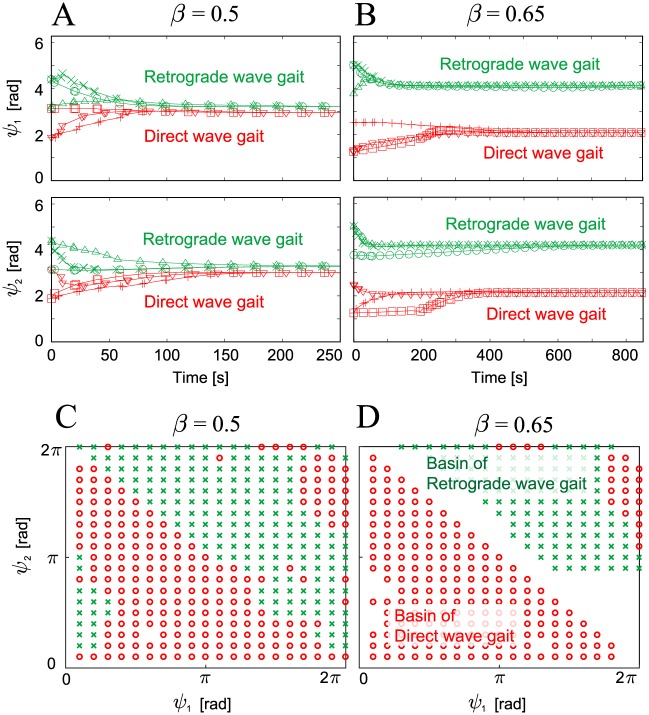
Relative phases (*ψ*_1_, *ψ*_2_) of the robot simulation plotted at the foot contact of Leg 2 and the basins of attraction. Relative phases are plotted for six initial conditions with (**A**) *β* = 0.5 and (**B**) *β* = 0.65. Six different markers represent the results for the six initial conditions. Irrespective of *β*, the robot established two different gaits (i.e., direct and retrograde wave gaits) that were dependent on the initial conditions. The basins of attraction for the two different gaits are plotted for (**C**) *β* = 0.5 and (**D**) *β* = 0.65. The red circles and green x points in (**C**) and (**D**) converge to the direct wave gaits and the retrograde wave gaits, respectively. The direct wave gaits have larger size of basins than the retrograde wave gait.

Fig 5A and 5B show the time profiles of the relative phases (*ψ*_1_, *ψ*_2_) for the six initial conditions for duty factors of *β* = 0.5 and 0.65, respectively. Data points are plotted when Leg 2 touches the ground (we use this condition for the Poincaré section). Depending on their initial relative phases, the phases converge to one of two different sets, irrespective of *β*. This means that there are two stable gaits: the direct and retrograde wave gaits. Fig 5C and 5D show the basins of attraction for the relative phases (*ψ*_1_, *ψ*_2_) for *β* = 0.5 and 0.65, where the red circles converge to the direct wave gait and the green x points converge to the retrograde wave gait. To calculate the basins, 400 lattice points are given on the relative phase plane as initial values and their convergence after 200 Poincaré mapping steps is examined. The direct wave gait has larger size of basins than the retrograde wave gait.

**Fig 6:**
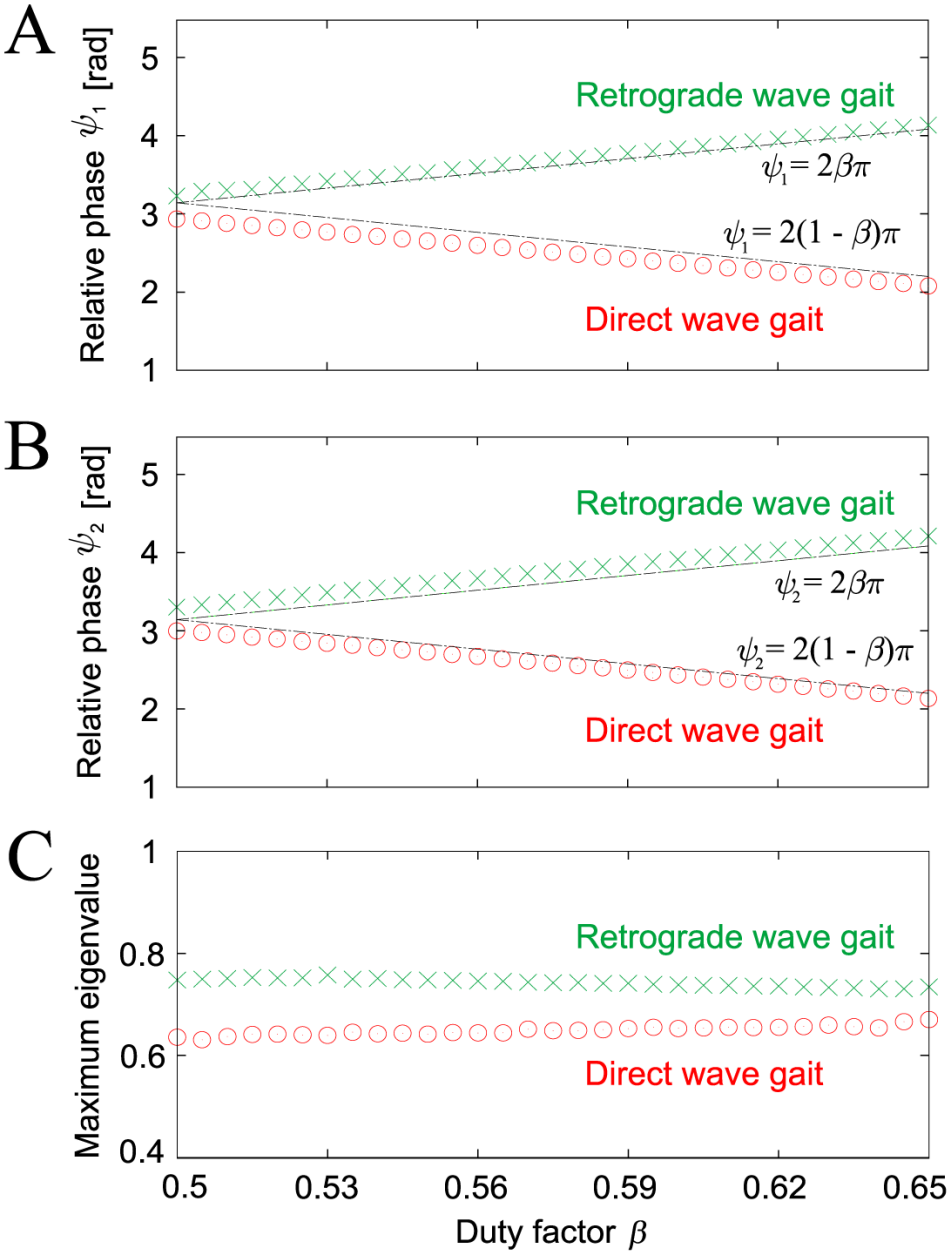
Relative phases and maximum eigenvalue of gaits obtained for duty factor *β* in computer simulations. **A**: Relative phase *ψ*_1_. **B**: Relative phase *ψ*_2_. **C**: Maximum eigenvalue. Two stable gaits were found for each duty factor (which were direct and retrograde wave gaits). The relative phases of each of the gaits changed smoothly with changing locomotion speed (duty factor *β*).

Fig 6A and 6B show the relative phases (*ψ*_1_, *ψ*_2_) of the converged gaits that were plotted when Leg 2 contacted the ground. Fig 6C shows the maximum absolute eigenvalue that was calculated based on a linear stability analysis of these gaits. These results show that our robot has two stable gaits (i.e., the direct and retrograde wave gaits), and that the relative phases of the two gaits change smoothly with locomotion speed, as per insect gaits (P2). We also note that the horizontal axis of Fig 1 (“1/Gait cycle”) is proportional to (1 − *β*) (see the Materials and Methods section).

One of the gaits obtained in the simulations is the direct wave gait, which satisfies the following phase relationship:

$$\begin{array}{c} \psi_{1}∼\psi_{2}∼2\left(1-\beta \right)\pi . \end{array}$$ (1)

**Fig 7:**
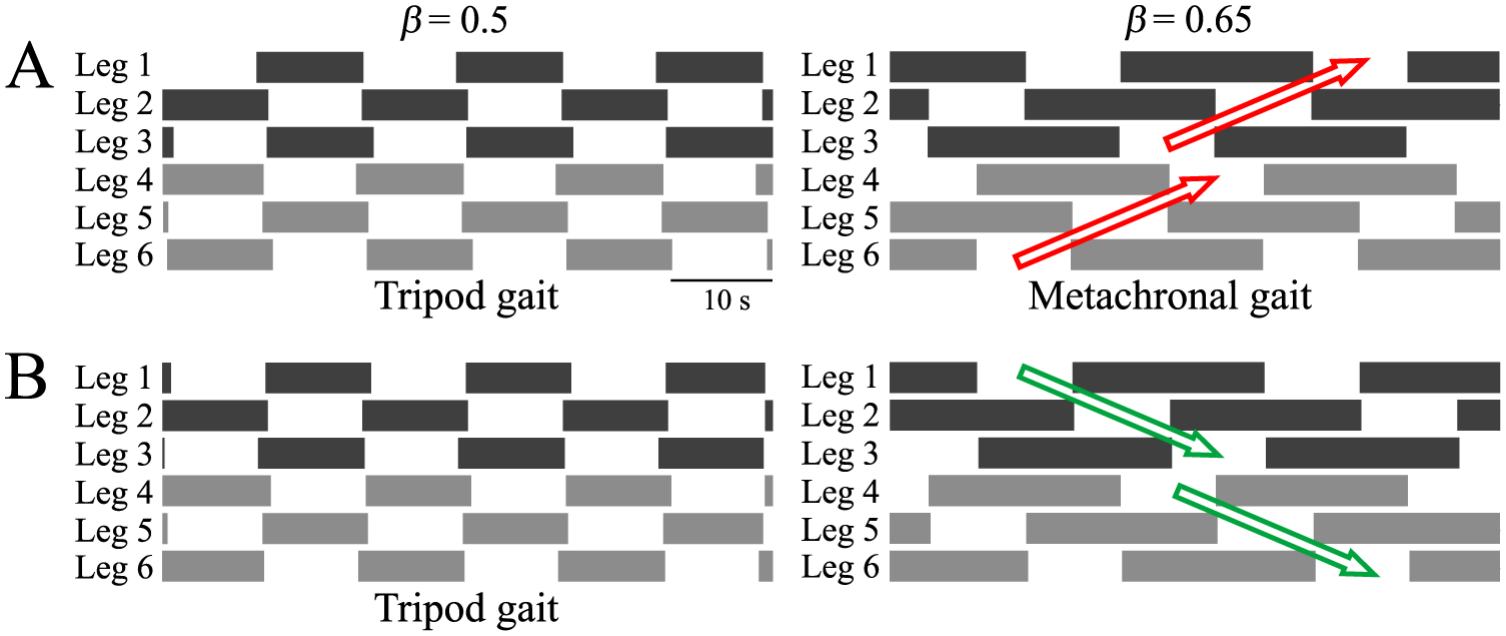
Footprint diagrams of the gaits obtained at duty factors of *β* = 0.5 and 0.65 in computer simulations. **A**: Direct wave gait. **B**: Retrograde wave gait.

This relationship is derived from Fig 6A and 6B. The red circles in these figures are close to the line *ψ* = 2(1 − *β*)*π*. In this gait, the swing movement of the legs propagates from posterior to anterior. The middle leg (the fore leg) lifts off just after the hind leg (the middle leg) touches the ground, as shown in Fig 7A. This gait therefore fulfils both insect gait properties P1 and P2. When *β* = 0.5, at least three legs are always in contact with the ground and the movements of these three legs are in phase, which means that this is a tripod gait. In contrast, when *β* = 0.65, at least four legs are always in contact with the ground, but the leg movements are not in phase. Because the swing movement of these legs propagates from posterior to anterior, this is a metachronal gait.

The other gait is the retrograde wave gait, which satisfies the following phase relationship:

$$\begin{array}{c} \psi_{1}∼\psi_{2}∼2\beta \pi . \end{array}$$ (2)

This relationship is derived from Fig 6A and 6B. The green x points in these figures are close to the line *ψ* = 2*βπ*. In this gait, the swing movement of the legs propagates from anterior to posterior and the middle leg (the hind leg) lifts off just after the fore leg (the middle leg) touches the ground, as shown in Fig 7B. This gait does not fulfil insect gait property P1. When *β* = 0.5, this corresponds to a tripod gait because at least three legs are always in contact with the ground and the movements of the three legs are in phase. However, when *β* = 0.65, while at least four legs are always in contact with the ground, the swing movement of the legs propagates from anterior to posterior, which differs from the metachronal gait.

In addition, the retrograde wave gait has smaller size of basins than the direct wave gait (Fig 5C and 5D). Furthermore, the retrograde wave gait has higher maximum eigenvalues than the direct wave gait in the Jacobian matrix of the Poincaré map (Fig 6C). This means that the retrograde wave gait tolerates smaller disturbances than the direct wave gait and that it takes more time for disturbances to vanish from the retrograde wave gait than for the direct wave gait.

### Robot experimental results

**Fig 8:**
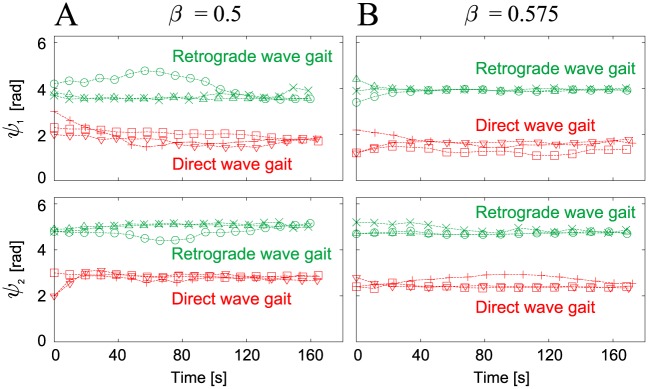
Relative phases (*ψ*_1_, *ψ*_2_) of the robot experiments plotted at foot contacts of Leg 2. Relative phases are plotted for six initial conditions with (**A**) *β* = 0.5 and (**B**) *β* = 0.575. The six different markers represent the results for the six initial conditions. Irrespective of the value of *β*, the robot established two different gaits that were dependent on the initial conditions.

To validate the simulation results above, we performed experiments using the hexapod robot (Fig 2A). We used various values for the duty factor *β* in the range from 0.5 to 0.65, and used six initial values for the relative phases (*ψ*_1_, *ψ*_2_) for each *β*. Fig 8A and 8B show the time profiles of the relative phases (*ψ*_1_, *ψ*_2_) that were plotted when Leg 2 touched the ground for duty factors of *β* = 0.5 and 0.575, respectively. Irrespective of the value of *β*, the relative phases converged to one of two different sets, which again means that there are two stable gaits. These two gaits correspond to the direct and retrograde wave gaits from the simulation results.

**Fig 9:**
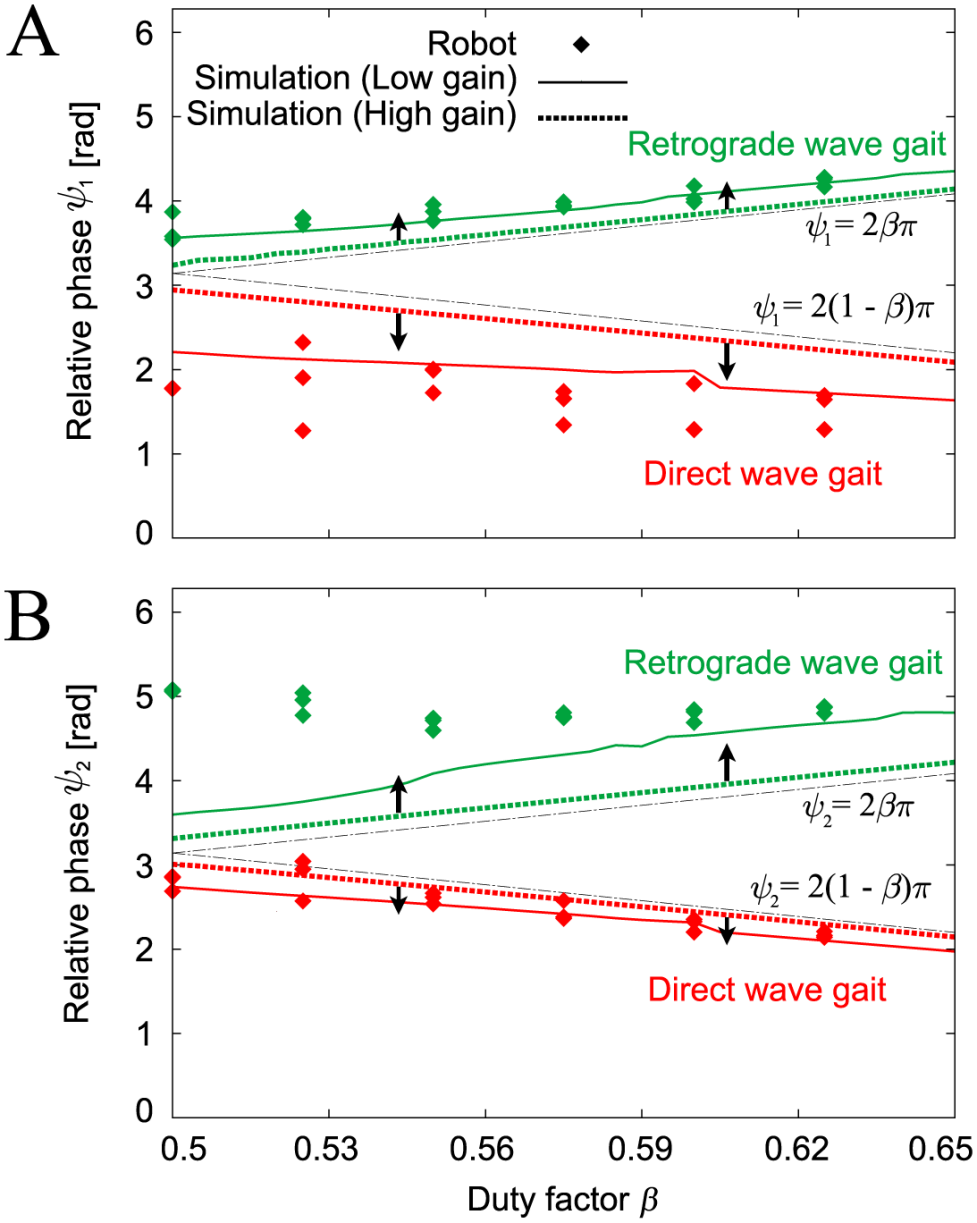
Relative phases (A) *ψ*_1_ and (B) *ψ*_2_ of the gaits obtained for duty factor *β* in the robot experiments and the computer simulations. Two stable gaits were obtained in the robot experiments: direct and retrograde wave gaits. The computer simulations used high and low feedback gains. When the feedback gain was reduced, the simulation results became much closer to the robot experimental results.

Fig 9 shows the relative phases (*ψ*_1_, *ψ*_2_) of the converged gaits for *β* where the data points of the robot experiments are the average values from three gait cycles after the robot walked for over two minutes for each trial. There are two different gaits (the direct and retrograde wave gaits) and the relative phases changed smoothly with changes in the locomotion speed, as shown in the simulation results in Fig 6A and 6B. The results for *ψ*_1_ for the direct wave gait and *ψ*_2_ for the retrograde wave gait differ slightly from the simulation results (dotted lines in Fig 9). To clarify the reasons for these differences, we performed computer simulations that involved reduction of the PD feedback gains of the joint controller. The feedback gain of the motor controller in our robot is low because of hardware limitations. The simulation results that corresponded to low gain feedback (indicated by the solid lines in Fig 9) were closer to the robot experimental results. While small differences still exist because of the limitations of the hardware, the robot experimental results are consistent with these simulation results. The direct and retrograde wave gaits of the robot for a duty factor of *β* = 0.6 are shown in the Supporting Information in S1 and S2 Movies, respectively.

### Analysis using the simple physical model

Our hexapod robot produced characteristic interlimb coordination based on the phase relationships among the ipsilateral oscillators that were dependent on the locomotion speed, despite the lack of direct interaction among the ipsilateral oscillators. This result emerged from the local sensory feedback, which was composed of phase resetting. To clarify the contribution of this embodied sensorimotor interaction to the determination of the phase relationship, we used a simple physical model of our hexapod robot and investigated its gait mechanism from a stability viewpoint. Here, we briefly explain the simple physical model. Full details are presented in the Materials and Methods section.

**Fig 10:**
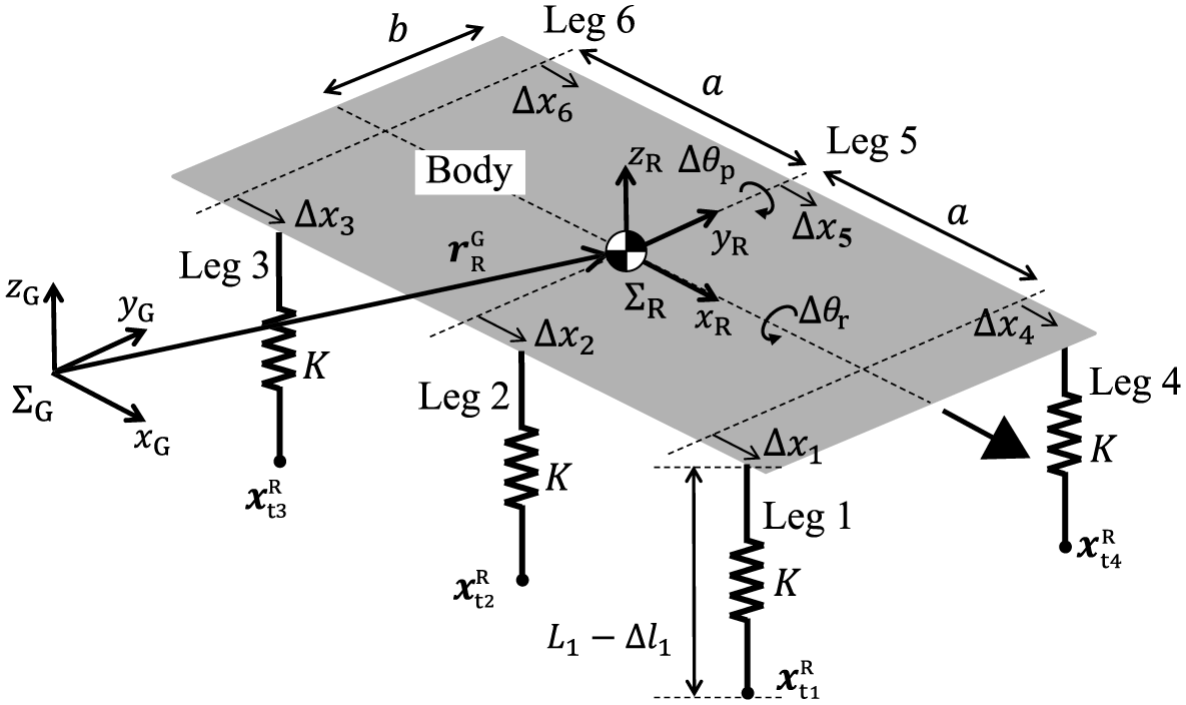
Simple physical model with rigid body and six massless spring legs. The body is represented by a flat plate here to show the geometric relationships between the model and the variables more clearly.

The simple physical model (Fig 10) is reduced from our hexapod robot model and the oscillator-based controller on the basis of certain physical assumptions. The model consists of a rigid body (mass: *M*; length: 2*a*; width: 2*b*) and six massless spring legs. The spring legs, which each have a spring constant of *K*, are vertically attached at the bottom of the body at intervals of *a* and represent the physical influence of the feedback controllers for the leg joints on the body (see assumption A1 in the Materials and Methods section). Based on the leg trajectory that was designed based on the oscillator phase *ϕ*_*i*_ (Fig 4), we determine the root position Δ*x*_*i*_ and the neutral length *L*_*i*_ of the spring using *ϕ*_*i*_. Because our robot walked with a long gait cycle (i.e., at low speed), we investigated this simple model using its static equilibrium. We then obtained approximate solutions and determined the stability of these solutions.

### Simple model analysis results

**Fig 11:**
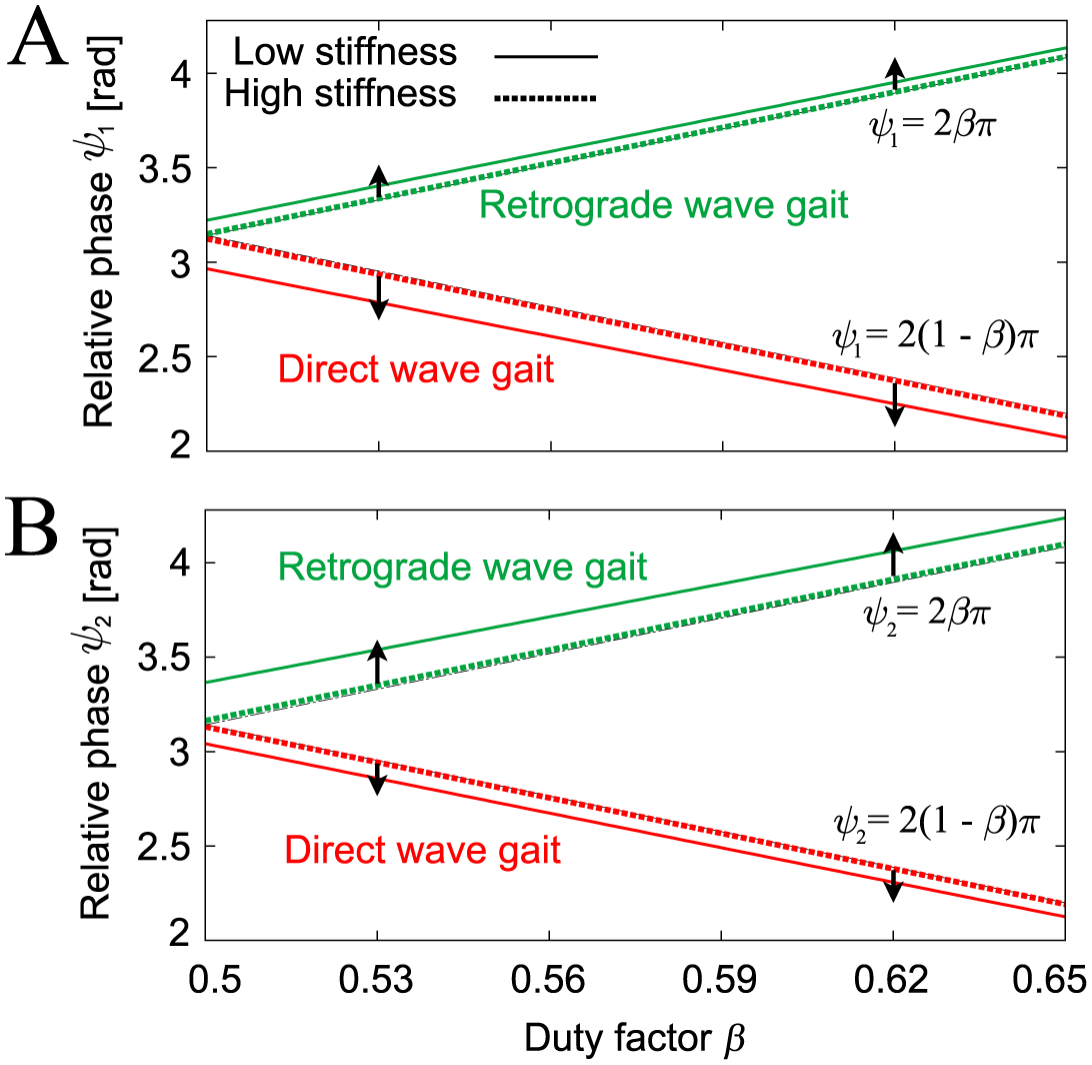
Relative phases A (*ψ*_1_) and B (*ψ*_2_) of the direct and retrograde wave gaits from the simple model. The relative phases are derived with both high stiffness (d* K* = 50)(solid line) and low stiffness (d* K* = 5)(dashed line) for s*/a* = 0.3. When the stiffness decreases, the relative phases *ψ*_1_ and *ψ*_2_ move away from 2*βπ* and 2(1 − *β*)*π* in a similar manner to the robot model in Fig 9. In addition, the relative phases of each gait change smoothly with changes in locomotion speed (duty factor *β*), as per the simulation.

We derived periodic solutions for the two different gaits (the direct and retrograde wave gaits). The fixed points in the Poincaré section, which corresponds to the touchdown points of Leg 2 without loss of generality, for these solutions are given by

$$\begin{array}{ccc} \psi_{1}^{\text{Dw}} & = & 2\left(1-\beta \right)\pi -2\left(1-\beta \right)\frac{1}{d^{*}K^{*}}+\frac{2}{5}\frac{1-\beta }{\beta }\frac{1}{d^{*}K^{*}}\frac{s^{*}}{a^{*}}+O\left({\left(K^{*}\right)}^{-2}\right),\\ \psi_{2}^{\text{Dw}} & = & 2\left(1-\beta \right)\pi -\frac{9}{10}\left(1-\beta \right)\frac{1}{d^{*}K^{*}}+\frac{1}{25}\left(\frac{11}{\beta }-30\right)\left(1-\beta \right)\frac{1}{d^{*}K^{*}}\frac{s^{*}}{a^{*}}+O\left({\left(K^{*}\right)}^{-2}\right), \end{array}$$ (3)

and

$$\begin{array}{ccc} \psi_{1}^{\text{Rw}} & = & 2\beta \pi +\frac{9}{10}\left(1-\beta \right)\frac{1}{d^{*}K^{*}}+\frac{1}{25}\left(\frac{11}{\beta }-30\right)\left(1-\beta \right)\frac{1}{d^{*}K^{*}}\frac{s^{*}}{a^{*}}+O\left({\left(K^{*}\right)}^{-2}\right),\\ \psi_{2}^{\text{Rw}} & = & 2\beta \pi +2\left(1-\beta \right)\frac{1}{d^{*}K^{*}}+\frac{2}{5}\frac{1-\beta }{\beta }\frac{1}{d^{*}K^{*}}\frac{s^{*}}{a^{*}}+O\left({\left(K^{*}\right)}^{-2}\right), \end{array}$$ (4)

where $\left(\psi_{1}^{\text{Dw}},\;\psi_{2}^{\text{Dw}}\right)$ and $\left(\psi_{1}^{\text{Rw}},\;\psi_{2}^{\text{Rw}}\right)$ are the fixed points of the direct and retrograde wave gaits, respectively. ()* indicates a dimensionless parameter (see the Materials and Methods section), and s* and d* are the dimensionless length and height shown in Fig 4B, respectively. Fig 11 shows these fixed points, which are consistent with the corresponding points in our robot simulations (Fig 6); *ψ*_1_ = *ψ*_2_ = 2*βπ* + *O*((K*)^−1^) for the direct wave gait and *ψ*_1_ = *ψ*_2_ = 2(1 − *β*)*π* + *O*((K*)^−1^) for the retrograde wave gait. In addition, these fixed points have similar dependences on the feedback gain (see Fig 9).

**Fig 12:**
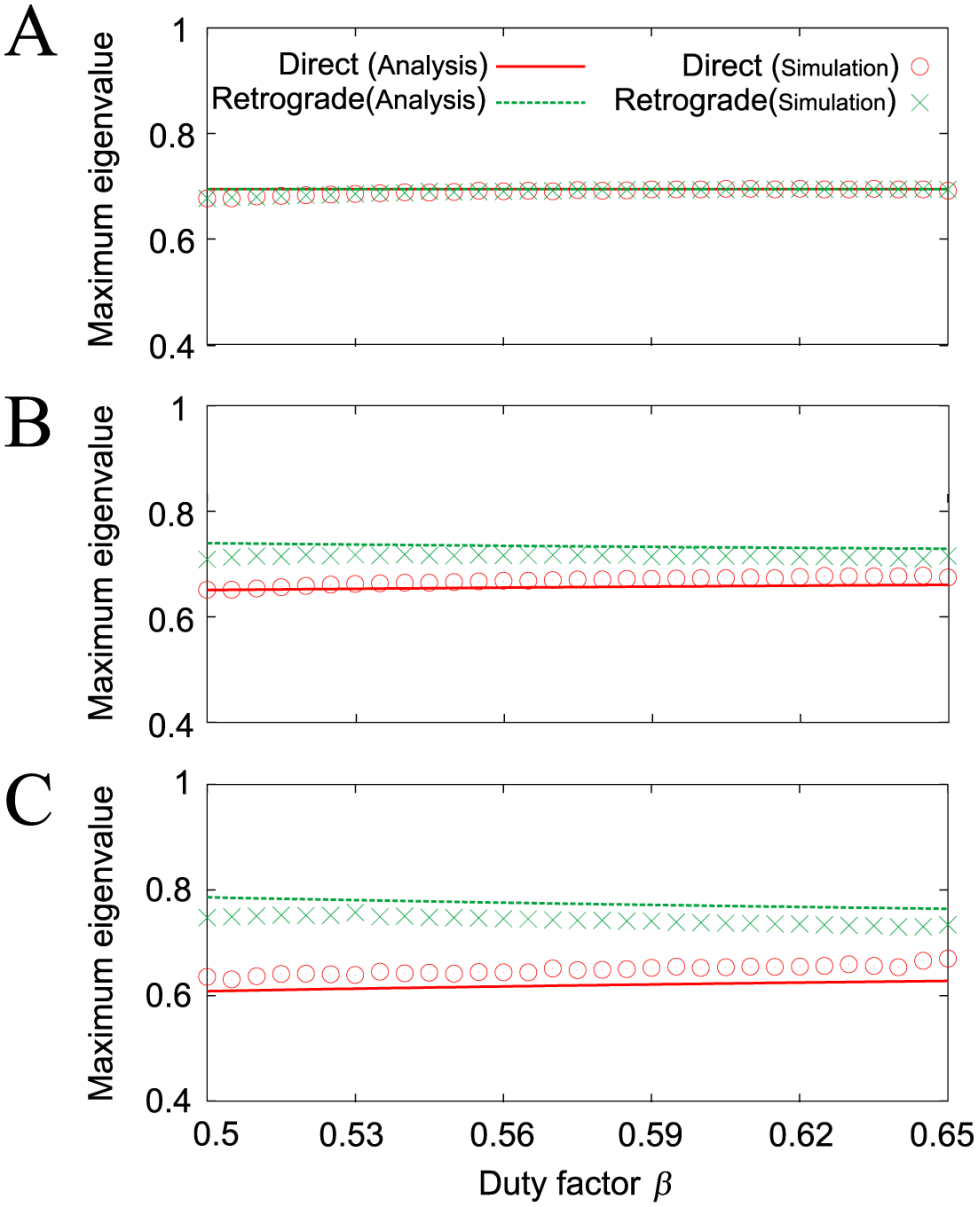
Maximum absolute eigenvalues of the direct and retrograde wave gaits of the simple model (Analysis) and the robot simulation (Simulation) for duty factor *β*. **A**: s*/a* = 0, **B**: 0.15, and **C**: 0.3.

Additionally, we obtained the maximum eigenvalues of the Jacobian matrix of the Poincaré map of (*ψ*_1_, *ψ*_2_) for these two gaits:

$$\begin{array}{ccc} \lambda^{\text{Dw}} & = & {\left(\frac{5}{6}-\frac{4}{45\beta }\frac{s^{*}}{a^{*}}\right)}^{2},\\ \lambda^{\text{Rw}} & = & {\left(\frac{5}{6}+\frac{4}{45\beta }\frac{s^{*}}{a^{*}}\right)}^{2}, \end{array}$$ (5)

where *λ*^Dw^ and *λ*^Rw^ represent the eigenvalues for the direct and retrograde wave gaits, respectively. The gait stability is dependent on s*/a* and these gaits are asymptotically stable for small values of s*/a*. While the two gaits have the same stability (*λ*^Dw^ = *λ*^Rw^) for s*/a* = 0, the direct wave gait is more stable than the retrograde wave gait (*λ*^Dw^ < *λ*^Rw^) for s*/a* > 0, as determined in the simulation results (Fig 6C). Fig 12 compares the maximum eigenvalues from the simple model with those from the robot simulation. The results of the simple model analysis and the robot simulation are clearly similar. Details of the derivation of these solutions and their stability are presented in the Materials and Methods section.

## Discussion

### Interlimb coordination generated by local sensory feedback

In this study, we have investigated the effects of local sensory feedback, in the form of phase resetting, on the interlimb coordination during hexapod locomotion using a minimalistic modeling approach. In contrast to similar previous approaches [31, 37–39], the ipsilateral oscillators in our model had no direct interactions. Our results show that our robot simulation model and the robot using the local sensory feedback generate two different gaits: direct and retrograde wave gaits. The direct wave gait is similar to an insect gait. In addition, the interlimb coordination of the two generated gaits changed smoothly, as observed in insect gaits (Fig 1B) [4, 5, 8, 11]. These gaits are not designed; instead, they emerge as a result of the embodied sensorimotor interaction. In addition, the simple model analysis replicates the results of both the robot simulations and experiments well, and the analysis also reveals the essence of the stability mechanism through analytical solutions. The model shows that these phenomena can happen when the walking speed is slow and the legs are elastic, as per physical assumptions A1-7 in the Materials and Methods section. These results indicate that local sensory feedback with phase resetting contributes to generation of ipsilateral interlimb coordination during hexapod locomotion, which is consistent with physiological indications [40, 41].

Some works have been performed to understand the interlimb coordination mechanism. For example, Cruse and colleagues [22–25] identified six rules to establish interlimb coordination based on behavioral studies, and proposed a bio-inspired controller using an artificial neural network called Walknet. This network created various movements for hexapod robots, including gait transitions, curve walking and searching behavior, as observed in stick insects. Daun-Gruhn [26] developed an oscillator network model of stick insect walking based on the use of CPGs for each leg joint and sensory feedback. This model had both excitatory and inhibitory synaptic connections for the oscillators for the ipsilateral front to rear legs. While these works achieved insect-like walking behavior, their models were complicated to allow clarification of the interlimb coordination mechanism. Our analytical expression gives a better understanding of the functional effects of foot contact sensory feedback for interlimb coordination.

Similar studies using quadruped robots proposed a simple local sensory feedback mechanism that used leg loading information [42–44], and showed that interlimb coordination of the type observed in quadruped animals emerges through embodied sensorimotor interactions. Owaki et al. [30] showed that the hexapod robot can generate insect like interlimb coordination solely by using the local and neighboring leg loading information designed by the Tegotae based approach. Their minimal model reproduced various insects’ gait pattern including the adaptation to leg amputation. Although the Tegotae approach and our approach use foot contact sensory feedback as local sensory information to achieve insect-like ipsilateral interlimb coordination, our approach relies only on the regulation of leg retraction timing while the Tegotae approach is based on a function that quantitatively measures a perceived reaction (i.e., sensor feedback) and an expectation (intention) of a controller which can be considered as an internal model. In addition, because of the simplicity of phase resetting, our simple model allows us to give an analytical explanation as to why the local sensory feedback determines the gaits in hexapods, which have not been explained in above studies (see the Materials and Methods section).

### Direct and retrograde wave gaits

Our robot simulation model and our robot generate both the direct and retrograde wave gaits using the local sensory feedback. In addition, the direct wave gait has a larger basin and a lower maximum eigenvalue than the retrograde wave gait. This means that perturbations in the direct wave gait disappear more rapidly than those in the retrograde wave gait and the direct wave gait can tolerate larger disturbances. Hughes [8] stated that at the liftoff of the fore legs, the center of mass (COM) in the retrograde wave gait within the supporting polygon is less than that in the direct wave gait. Our results suggest that the direct wave gait is better for robust walking, as proposed by Hughes.

In addition, the main reason why the stabilities of the two gaits are different is determined via the simple model analysis, as shown in the Materials and Methods section. The analysis results indicate that the retrograde wave gait is more stable than the direct wave gait when the model walks in the backward direction, which shows that the position of the COM relative to the supporting polygon affects the stability of the gait through embodied sensorimotor interaction.

### Smooth and discontinuous gait transitions

While some quadruped animals such as dogs can change their gaits discontinuously depending on their locomotion speed, as shown in Fig 1A, other quadruped animals such as sheep, and certain insects, such as stick insects, change their gaits smoothly, as shown in Fig 1B. Schöner et al. [45] suggested that these gaits are the result of self-organization in a complex dynamic system. From this perspective, the differences between smooth and discontinuous gait transitions can be explained in terms of the gait stability structures.

Discontinuous gait transitions indicate that only some parts of the interlimb coordination can exist stably, and that these parts are separated. The appearance of hysteresis within the gait transition reflects this stability structure [33, 46]. In previous work [32], we used a simple quadruped model and an oscillator network with phase resetting to show that saddle-node bifurcations induce discontinuous gait transitions and hysteresis using a dynamic stability analysis.

In contrast, smooth gait transitions indicate that all interlimb coordination within a specific range can exist stably. In this study, we show that the change in the gait of our robot simulation model occurs smoothly and is dependent on the locomotion speed (Fig 6A and 6B), as observed in stick insects (Fig 1B). In the case of the direct wave gait, the model generates a metachronal gait at slow speeds (*β* = 0.65), and this gait transits smoothly to a tripod gait (*β* = 0.5) as the speed increases. This dependence of the gait on the locomotion speed can be explained via an analysis of the static stability of the body dynamics using our simple model, as shown in Fig 11. These results indicate that the discontinuous gait transition mechanism arises from dynamic stability, while the smooth gait transition mechanism can be explained based on static stability.

### Role of sensory feedback in fast and slow locomotion

It has previously been suggested that sensory feedback does not play a primary role in high–speed locomotion [47]. For example, the high–speed walking motions of cockroaches were analyzed using a simple planar model that was composed of a rigid body with massless spring legs [48, 49], and the results showed that self-stabilization based on intrinsic musculoskeletal properties makes a greater contribution to the generation of locomotion than the sensory feedback.

Conversely, it has also been suggested that sensory feedback plays a critical role in low–speed locomotion, as observed in stick insects [47, 50]. For example, a neuromechanical model of a stick insect leg showed that the three leg joints were all controlled by independent bistable neural circuits with sensory feedback [51]. In addition, computer simulations and robot experiments involving low–speed movement demonstrated that coordinated leg joint movements are generated by neuromechanical interactions through sensory feedback [52]. Some studies proposed use of positive feedback of the angular velocity for joint control [24, 53], which contributes to the adjustment of the leg trajectory and thus reduces mechanical stress [28]. Our model focuses on the embodied sensorimotor interactions produced by local sensory feedback to clarify the mechanisms of low–speed insect gaits.

### Limitations and future work

Because we used a minimalistic modeling approach, there are obviously differences between our model and actual insects. For example, while we assumed that the left and right oscillators were in antiphase, there is no evidence to date of strong coupling of the left and right leg pairs in insects [6, 54]. In addition, it has also been reported that insect gaits cannot be identified unequivocally depending on individual situations [54]. In particular, when insects walk backwards, they do not tend to show well-coordinated gaits [55]. In addition, it has been reported that stick insects can achieve interlimb coordination even if their body postures are fixed dorsally to a holder [56]. Our model cannot explain this behavior because our model achieves interlimb coordination through regulation of the leg retraction timings, which can vary according to changes in the body posture. While we did succeed in clarifying the effects of sensory feedback on ipsilateral interlimb coordination, the above properties cannot be explained based on the focused sensory feedback alone. We would therefore like to analyze these properties in future by considering other types of sensory feedback, e.g., joint angle feedback.

Physically, the mass and size of the robot are unlike the corresponding properties of insects. In addition, the robot’s joints are controlled by high–gain servo motors, while insects use muscles to control their joints. In the neural model, we used a simple CPG-based controller and a sensory feedback model. However, our simple model and the associated analysis can provide meaningful insights into both the biological sciences and engineering, as many studies have shown [57–60]. In particular, because our simple model extracted the essential components that are required for hexapod static walking, it can provide a basis for further analysis of insect gaits and offer hints for adaptive walking design. For example, the constraint that has been imposed between the left and right oscillators can easily be removed from our simple model for the purposes of further analysis. Additionally, other gait types that we did not analyze in this work, such as the tetrapod gait [54], can also be investigated more easily using our simple model than through use of a complex insect model. Sensory feedback with leg loading information can also be applied to our simple model. This simple model will also be helpful in the design of a distributed control method for legged robots that can adapt to leg amputation, because the model is simple to formulate. In addition, our analysis can be extended to multi-legged and quadruped models. The direct wave gait has also been observed in quadrupeds and millipedes. Conversely, the retrograde wave gait has also been observed in some centipedes. It will also be possible to analyze the effects of sensory feedback on these gaits by simply extending our analysis. In the future, we will test on uneven ground as well as investigate mechanisms underlying continuous and discontinuous gait transitions.

## Materials and methods

### Hexapod robot

The hexapod robot (AMOS II [17] in Fig 2) is composed of a single body and six legs (Legs 1–6). Each leg consists of three links (Links 1–3), which are connected using joints that are controlled by servo motors (Joints 1–3). Joint 1 is a yaw joint that moves the leg from back to front. Joints 2 and 3 are the pitch joints that lift the leg up and down. A touch sensor has been installed on the tip of each leg. Table 1 lists the physical parameters of the robot in the case where all six legs are identical.

**Table 1:** Physical parameters of the robot.

Link	Parameter	Value
Body	Mass [kg]	4.6
	Width [mm]	100
	Length [mm]	400
Leg link 1	Mass [kg]	0.27
	Length [mm]	65
Leg link 2	Mass [kg]	0.27
	Length [mm]	65
Leg link 3	Mass [kg]	0.27
	Length [mm]	115

The robot is battery-powered and is controlled using an external host computer (central processing unit (CPU): Intel Core i5 2.5 GHz; memory: 8 GB) with commands sent at 1/30 s intervals. Communications between the robot and the computer are conducted via the serial interface. The serial cable remains slack so that it does not affect the locomotion of the robot. For computer simulation of our hexapod robot model, we used the LPZROBOTS computer simulator, which is based on the Open Dynamics Engine (ODE) [61].

### Controller

**Phase oscillator and motor control** The phase oscillators follow these dynamics:

$$\begin{array}{ccc} \dot{\phi_{i}} & = & \omega +g_{i}+y_{i}, \end{array}$$ (6)

$$\begin{array}{ccc} \tau {\dot{y}}_{i} & = & k_{\text{f}}^{i}-y_{i}, \end{array}$$ (7)

where *ω* is the basic locomotion frequency and *g*_*i*_ is the interaction between the oscillators (see the section below entitled Hexapod gait in the phase oscillator model). $k_{\text{f}}^{i}$ represents the sensory feedback, which is incorporated in a first-order lag system using *y*_*i*_ and the time factor *τ* (= 1 s) (see the section below entitled Sensory feedback).

For motor control, the tip of Leg *i* follows a trajectory relative to the body that is based on *ϕ*_*i*_ (Fig 4). During the stance phase (0 ≤ *ϕ*_*i*_ < 2*βπ*), the leg tip moves along a line segment that runs between the anterior extreme position (AEP) and the posterior extreme position (PEP), which lies parallel to the body. During the swing phase (2*βπ* ≤ *ϕ*_*i*_ < 2*π*), the leg tip moves along a simple ellipsoid curve that includes both the AEP and the PEP. *β* is the duty factor (i.e., the ratio of the stan phase and step cycle durations). We used the distance between the AEP and PEP, denoted by *s* = 6 cm, and the height of the ellipsoid, denoted by *d* = 6 cm. Each joint was controlled using a PD feedback controller to generate the desired joint angle that was calculated using inverse kinematics.

We set the duration of the swing phase to be *T*_sw_ = const., as is often observed in insects [4, 5]. The step cycle duration *T*_p_, the basic frequency *ω* in (6), the stride length *S*_l_ and the locomotion speed *v* are then given by *T*_p_ = *T*_sw_/(1 − *β*), *ω* = 2(1 − *β*)*π*/*T*_sw_, *S* = *s*/*β*, and *v* = (1 − *β*)*s*/*βT*_sw_, respectively, i.e., they are all determined based on *β*. We set *T*_sw_ = 5 s in the experiments and 10 s in the simulations.

**Hexapod gait in the phase oscillator model** Because the leg movements of our robot are determined by the oscillation phases, the relative phases between the oscillators must explain the gait, which are produced by interactions among the contralateral oscillators and the sensory feedback.

In insect gaits, the ipsilateral phase relationships change depending on the speed of motion, while the contralateral phase relationships are almost in antiphase [5]. To ensure a simple system, we assume that the contralateral legs alternate in phase. Therefore, the interactions between the oscillators *g*_*i*_ in (6) are described as follows (Fig 3):

$$\begin{array}{c} g_{i}=-\sum_{j=1}^{6}k_{\text{c}}^{ij}\sin\left(\phi_{i}-\phi_{j}-\pi \right), \end{array}$$ (8)

where

$$\begin{array}{ccc} k_{\text{c}}^{ij} & = & \left\{ \begin{array}{cc} k_{\text{c}} & \left(i,\;j)\in \{(1,4),(2,5),(3,6),(4,1),(5,2),(6,3)\right\}\\ 0 & \text{otherwise}. \end{array}\right. \end{array}$$

We used a large value for *k*_c_ (= 10) so that the relative phases between the left and right oscillators are constrained to values of *π*. There is no direct interaction that could produce another relationship among the oscillators (ipsilateral coordination).

Because the relative phases between the left and right legs are set in antiphase, the gait in our phase oscillator model can be explained using the two relative phases *ψ*_1_(= *ϕ*_2_ − *ϕ*_1_) and *ψ*_2_(= *ϕ*_3_ − *ϕ*_2_), which are determined based on locomotion dynamics.

**Sensory feedback** Sensory feedback plays an important role in determining the coordinated motor outputs of the CPGs during locomotion [14, 50, 62–65]. Physiological evidence has shown that detection of an increasing load on the leg promotes leg retraction [34, 35], and some interneurons can cause a reset of the rhythmicity during motoneuron activities [36]. The motor outputs of the CPGs are thus modulated by phase shifting and rhythm resetting based on foot contact information (phase resetting).

Based on these findings, we incorporated the phase resetting mechanism from our previous work [31] and thus determined $k_{\text{f}}^{i}$ in (7) by

$$\begin{array}{c} k_{\text{f}}^{i}=\left\{ \begin{array}{cc} 0 & 0\leq \phi_{i}\left(t_{\text{o}}^{i}\right)<2\beta \pi \\ \left\{2\pi -\phi_{i}\left(t_{\text{o}}^{i}\right)\right\}\delta \left(t-t_{\text{o}}^{i}\right) & 2\beta \pi \leq \phi_{i}\left(t_{\text{o}}^{i}\right)<2\pi , \end{array}\right. \end{array}$$ (9)

where $t_{\text{o}}^{i}$ is the time when Leg *i* touches the ground and *δ*() is the Dirac delta function. When Leg *i* touches the ground during the swing phase (2*βπ* ≤ *ϕ*_*i*_ < 2*π*), as indicated by point R in Fig 4A, the phase *ϕ*_*i*_ is then reset to zero. We denote $\phi_{i}\left(t_{\text{o}}^{i}\right)$ here as $\phi_{i}^{\text{td}}$ (i.e., the touchdown phase). In this paper, we used a first-order lag system with time factor *τ* to vary the phase value continuously [66] for the robot simulation model. Because of this phase resetting process, leg load detection triggers retraction of the leg. This can be regarded as a simplified description of the sensory feedback process in insects that was described above [34, 35].

### Simple physical model

**Physical assumptions** To clarify the underlying mechanisms that allow our hexapod robot to produce two different gaits and to change the phase relationship between the tripod and metachronal gaits smoothly with changes in the locomotion speed, we develop a simple physical model (Fig 10) based on the following assumptions:

A1 Because the legs of the robot are much lighter than its body, we neglect the mass of its legs. We also replace the physical influence of the PD feedback controllers of the leg joints on the body through use of spring legs. The angular displacements from the commanded angles in the joints yield forces that are proportional to these displacements because of the PD feedback controllers in the robot simulation model and in the robot. We therefore model this effect simply using the springy leg in the simple model. Specifically, we use six massless springs (with spring constant *K*) that are vertically attached (spaced at interval *a*) to the bottom of the body (mass: *M*; length: 2*a*; width: 2*b*). Touchdown and liftoff both occur at the neutral length and the springs only work during the stance phase.
A2 Because the gait cycle of our robot was more than 20 s, which ensures that the robot’s gait is static, we investigated the static equilibrium while neglecting the horizontal friction that occurs between the leg tips and the ground.
A3 Because the leg trajectory was designed to ensure that our robot walks in a straight line (Fig 4B), we have neglected the yaw motion.
A4 Based on the leg trajectory that was designed based on the oscillator phase *ϕ*_*i*_, we determine the root position Δ*x*_*i*_ and the neutral length *L*_*i*_ of the spring using *ϕ*_*i*_ (Δ*x*_*i*_ = Δ*x*_*i*_(*ϕ*_*i*_), *L*_*i*_ = *L*_*i*_(*ϕ*_*i*_)). We also assume that the toe position can be changed without any dynamics.
A5 Because the feedback gains of our robot are large enough to follow the desired leg trajectory, particularly in the computer simulations, we used a large value for the spring constant *K* and use this constant as an order parameter in the stability analysis.
A6 Because the relative phases of the left and right oscillators are constrained to a value of *π* in (6), we use *ϕ*_*i*+3_ = *ϕ*_*i*_ + *π*(*i* = 1, 2, 3).
A7 Because the time constant *τ* in (7) is much shorter than the gait cycle, we neglect the delay in the sensory feedback process (*τ* = 0).

In the simple model, we use the inertial frame *Σ*_G_(*x*_G_, *y*_G_, *z*_G_), which is fixed on the ground, and the robot coordinate frame *Σ*_R_(*x*_R_, *y*_R_, *z*_R_), which is fixed on the body, with an origin that is located at the COM. ***q***^G^ and ***q***^R^ are the vectors on *Σ*_G_ and *Σ*_R_, respectively. *x*_R_ is the walking direction of the model and *z*_G_ is the vertical direction. The robot posture is represented by the pitch angle Δ*θ*_p_ and the roll angle Δ*θ*_r_. We denote the position of the COM by $r_{\text{R}}^{\text{G}}$ on *Σ*_G_ and the position of the tip of Leg *i* by $x_{\text{t}i}^{\text{R}}$ on *Σ*_R_. The length of Leg *i* is represented by *L*_*i*_ − Δ*l*_*i*_, where Δ*l*_*i*_ is the compression. The displacement of the root of Leg *i* in the *x*_R_ direction is represented by Δ*x*_*i*_.

The positions of each of the leg tips $x_{\text{t}i}^{\text{R}}\left(i=1,\;\ldots ,\;6\right)$ on *Σ*_R_ are given by

$$\begin{array}{c} \left\{ \begin{array}{c} x_{\text{t}1}^{\text{R}}={\left[a+\Delta x_{1},\;\;\;-b,\;\;\;-\left(L_{1}-\Delta l_{1}\right)\right]}^{\text{T}}\\ x_{\text{t}2}^{\text{R}}={\left[\Delta x_{2},\;\;\;-b,\;\;\;-\left(L_{2}-\Delta l_{2}\right)\right]}^{\text{T}}\\ x_{\text{t}3}^{\text{R}}={\left[-a+\Delta x_{3},\;\;\;-b,\;\;\;-\left(L_{3}-\Delta l_{3}\right)\right]}^{\text{T}}\\ x_{\text{t}4}^{\text{R}}={\left[a+\Delta x_{4},\;\;\;b,\;\;\;-\left(L_{4}-\Delta l_{4}\right)\right]}^{\text{T}}\\ x_{\text{t}5}^{\text{R}}={\left[\Delta x_{5},\;\;\;b,\;\;\;-\left(L_{5}-\Delta l_{5}\right)\right]}^{\text{T}}\\ x_{\text{t}6}^{\text{R}}={\left[-a+\Delta x_{6},\;\;\;b,\;\;\;-\left(L_{6}-\Delta l_{6}\right)\right]}^{\text{T}}. \end{array}\right. \end{array}$$ (10)

Based on the leg tip trajectory of our robot shown in Fig 4B, the neutral length of the leg spring *L*_*i*_ and the displacement of the associated root Δ*x*_*i*_ are given as functions of the oscillator phase *ϕ*_*i*_, as follows:

$$\begin{array}{c} L_{i}=\left\{ \begin{array}{cc} L & 0\leq \phi_{i}<2\beta \pi \\ L-d\sin\frac{\phi_{i}-2\beta \pi }{2(1-\beta )} & 2\beta \pi \leq \phi_{i}<2\pi , \end{array}\right. \end{array}$$ (11)

$$\begin{array}{c} \Delta x_{i}=\left\{ \begin{array}{cc} s(\frac{1}{2}-\frac{\phi_{i}}{2\beta \pi }) & 0\leq \phi_{i}<2\beta \pi \\ s(-\frac{1}{2}+\frac{\phi_{i}-2\beta \pi }{2(1-\beta )\pi }) & 2\beta \pi \leq \phi_{i}<2\pi , \end{array}\right. \end{array}$$ (12)

where *L* is the neutral spring length during the stance phase.

To clarify the parameter dependence of the gait stability, we normalized the physical parameters. Specifically, we normalized the length parameter *p* with respect to *L* as p* = *p*/*L* and used the relative spring constant as given by K* = *KL*/*Mg*, where ()* indicates a dimensionless parameter. We assume the orders of the dimensionless parameters used for the stability analysis as follows:

$$\begin{array}{c} a^{*},\;b^{*},\;d^{*}≃O\left(1\right), \end{array}$$ (13)

$$\begin{array}{c} s^{*}≲O\left({\left(K^{*}\right)}^{-\frac{2}{3}}\right), \end{array}$$ (14)

$$\begin{array}{c} \Delta l_{i}^{*},\;\Delta \theta_{\text{p}},\;\Delta \theta_{\text{r}}≃O\left({\left(K^{*}\right)}^{-1}\right), \end{array}$$ (15)

We neglect *O*((K*)^−2^) here. However, the inequality of (14) means that we do not neglect the dimensionless parameters {Δp*, s*, (s*)^2^, s*Δp*} for Δp* ≃ *O*((K*)^−1^).

**Phase description of the model position and posture based on equilibrium of force and moment** When the vertical distance from the leg root to the ground is less than the neutral length $L_{i}^{*}$, and the compression of the leg spring $\Delta l_{i}^{*}\geq 0$, the leg is in contact with the ground. Otherwise, the leg must be in the air. Let *S* = {*i* ∣ Leg *i* on the ground} be the set of stance legs. When Leg *i* is in contact with the ground, the following constraint applies:

$$\begin{array}{c} {\left(x_{\text{t}i}^{\text{G}*}\right)}_{z}={\left(R_{\text{R}}^{\text{G}}x_{\text{t}i}^{\text{R}*}\right)}_{z}=0\;\;\;\;\;\;i\in S, \end{array}$$ (16)

where ()_*z*_ indicates the *z* element and the matrix $R_{\text{R}}^{\text{G}}$ is the approximate rotation matrix from *Σ*_R_ to *Σ*_G_ given by

$$\begin{array}{c} R_{\text{R}}^{\text{G}}=\left[\begin{array}{ccc} 1 & 0 & \Delta \theta_{\text{p}}\\ 0 & 1 & -\Delta \theta_{\text{r}}\\ -\Delta \theta_{\text{p}} & \Delta \theta_{\text{r}} & 1 \end{array}\right]. \end{array}$$

The constraint of (16) is approximated here using the dimensionless height h* ($={\left(r_{\text{R}}^{\text{G}*}\right)}_{z}$) as

$$\begin{array}{c} \left\{ \begin{array}{c} \Delta l_{1}^{*}=\left(a^{*}+\Delta x_{1}^{*}\right)\Delta \theta_{\text{p}}+b^{*}\Delta \theta_{\text{r}}+L_{1}^{*}-h^{*}\\ \Delta l_{2}^{*}=\Delta x_{2}^{*}\Delta \theta_{\text{p}}+b^{*}\Delta \theta_{\text{r}}+L_{2}^{*}-h^{*}\\ \Delta l_{3}^{*}=\left(-a^{*}+\Delta x_{3}^{*}\right)\Delta \theta_{\text{p}}+b^{*}\Delta \theta_{\text{r}}+L_{3}^{*}-h^{*}\\ \Delta l_{4}^{*}=\left(a^{*}+\Delta x_{4}^{*}\right)\Delta \theta_{\text{p}}-b^{*}\Delta \theta_{\text{r}}+L_{4}^{*}-h^{*}\\ \Delta l_{5}^{*}=\Delta x_{5}^{*}\Delta \theta_{\text{p}}-b^{*}\Delta \theta_{\text{r}}+L_{5}^{*}-h^{*}\\ \Delta l_{6}^{*}=\left(-a^{*}+\Delta x_{6}^{*}\right)\Delta \theta_{\text{p}}-b^{*}\Delta \theta_{\text{r}}+L_{6}^{*}-h^{*}, \end{array}\right. \end{array}$$ (17)

where the equation for $\Delta l_{i}^{*}$ is only applicable when *i* ∈ *S*. Based on this constraint, $\Delta l_{i}^{*}$ can be determined using Δ*θ*_r_, Δ*θ*_p_, h*, and *ϕ*_*i*_.

The ground reaction force is given by the sum of the spring compression forces of the stance legs, which is equivalent to the gravitational force, and thus yields the following equation:

$$\begin{array}{c} \sum_{i\in S}K^{*}\Delta l_{i}^{*}=1. \end{array}$$ (18)

In addition, the equilibria of the moments around the COM in the pitch and roll directions are approximated as follows:

$$\begin{array}{c} \sum_{i\in S}K^{*}\Delta l_{i}^{*}{\left(R_{\text{R}}^{\text{G}}x_{\text{t}i}^{\text{R}*}\right)}_{x}=0, \end{array}$$ (19)

$$\begin{array}{c} \sum_{i\in S}K^{*}\Delta l_{i}^{*}{\left(R_{\text{R}}^{\text{G}}x_{\text{t}i}^{\text{R}*}\right)}_{y}=0, \end{array}$$ (20)

where ()_*x*_ and ()_*y*_ indicate the *x* and *y* elements, respectively. From (18), (19), and (20), Δ*θ*_r_, Δ*θ*_p_, and h* can be determined using the oscillator phase *ϕ*_*i*_ with the dimensionless parameters a*, b*, d*, s*, and K*.

**Phase dynamics** Based on assumptions A6 and A7, the phase dynamics of (6) can be reduced to

$$\begin{array}{c} \dot{\phi_{i}}=\omega +\frac{1}{2}k_{\text{f}}^{i}+\frac{1}{2}k_{\text{f}}^{i+3}\;\;\;\;\;\;\;i=1,2,3, \end{array}$$ (21)

where $k_{\text{f}}^{i+3}$ indicates the sensory feedback from the leg on the opposite side and the coefficient 1/2 for both $k_{\text{f}}^{i}$ and $k_{\text{f}}^{i+3}$ comes from assumption A6 (a detailed explanation is presented in S1 Appendix in the supplementary file).

The sensory feedback $k_{\text{f}}^{i}$ only works at the foot contact. Because the model position h* and the posture (Δ*θ*_r_, Δ*θ*_p_) are represented by *ϕ*_*i*_, $k_{\text{f}}^{i}$ in (9) is explained using *ϕ*_*i*_. The state variables in this system are therefore summarized by *ϕ*_1_, *ϕ*_2_, and *ϕ*_3_, and the gait is then represented by the relative phases *ψ*_1_(= *ϕ*_2_ − *ϕ*_1_) and *ψ*_2_(= *ϕ*_3_ − *ϕ*_2_).

**Single constraint on the phase relationship immediately before a touchdown event** In this model, when the leg touches the ground, the vertical distance from the leg root to the ground is equal to the neutral length ($\Delta l_{i}^{*}=0$). Because the model position and posture are described using *ϕ*_*i*_, this equality gives only a single constraint for *ϕ*_*i*_.

### Derivation of periodic solutions and their stabilities

In this section, we derive periodic solutions for the direct and retrograde wave gaits and investigate the stability of these solutions through linear stability analysis. First, we deal with the direct wave gait, and then deal with the retrograde wave gait based on the symmetry properties of these gaits.

In one gait cycle, each leg experiences the swing and stance phases once. Because the relative phases between the oscillators change only at the moment of foot contact by phase resetting, as in (21), the reset value must be identical for all the oscillators for the periodic solution. This means that the phase value immediately before foot contact must be identical for all the oscillators (i.e., $\phi_{1}^{\text{td}}=\phi_{2}^{\text{td}}=\phi_{3}^{\text{td}}$).

**Fig 13:**
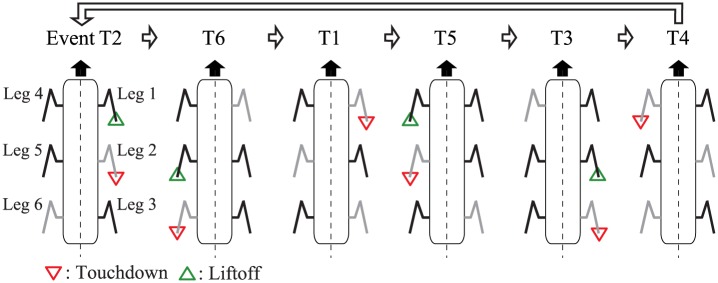
Touchdown and liftoff events for the direct wave gait. Black and grey legs represent the stance and swing legs, respectively. Events T*i* and T(*i* + 3) (*i* = 1, 2, 3) have axial symmetry.

**Direct wave gait** Fig 13 shows the sequence of the touchdown and liftoff events for the legs with the direct wave gait in the range around 1/2 < *β* < 2/3. The touchdown event of Leg *i* is denoted by event T*i*. Events T2, T6, T1, T5, T3, and T4 thus occur in that order for a single gait cycle. Because of the right and left symmetries of the simple model and the antiphase relationship between the left and right oscillators, our model thus has axial symmetry. Because the amount of phase resetting is determined by the geometric conditions at each event ($\Delta l_{i}^{*}=0$), the amount of phase resetting that occurs at event T*i* is made to be equivalent to that at event T(*i* + 3) by shifting each oscillator phase by *π*. Because the relative phases are only influenced by the amount of phase resetting that occurs at each event, as in (21), and because the phase shift by *π* does not affect the relative phases, we can then assume that events T4, T5, and T6 are equivalent to events T1, T2, and T3, respectively. Therefore, we investigate the sequence T2, T3, T1, T2, T3, and T1 for a single gait cycle, which means that we only need to examine half of the repeating events: T2, T3, and T1.

We denote the sets of stance legs immediately before event T*i*(*i* = 1, 2, 3) by $S_{\text{T}i}$, which is based on the relative phases *ψ*_1_ ∼ *ψ*_2_ ∼ 2(1 − *β*)*π* as

$$\begin{array}{c} S_{\text{T1}}=\left\{2,4,6\right\},\;S_{\text{T2}}=\left\{1,3,4,5\right\},\;S_{\text{T3}}=\left\{1,2,5,6\right\}. \end{array}$$ (22)

The details of these sets are presented in S2 Appendix of the supplementary file.

**Fig 14:**
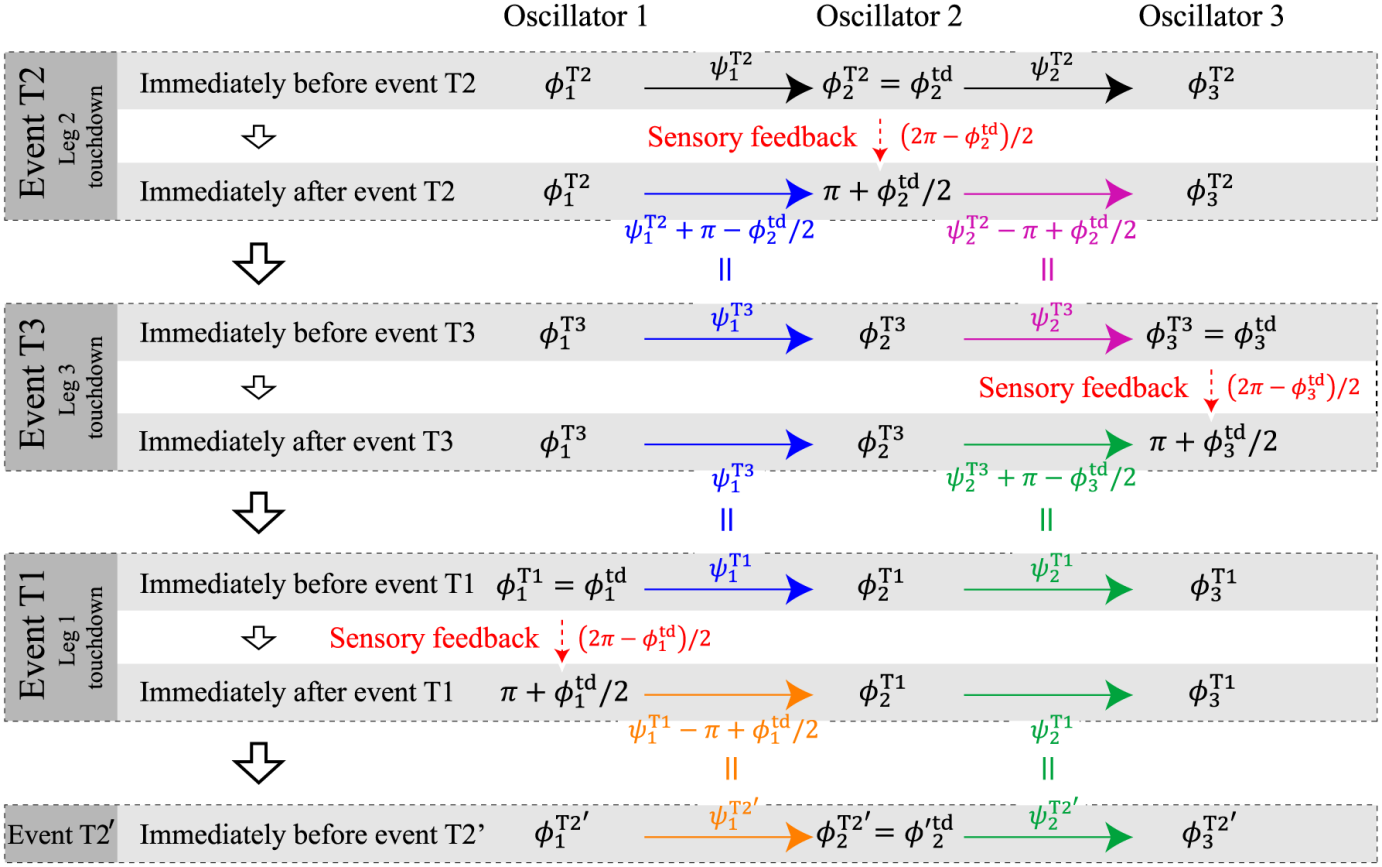
Evolution of the oscillator phases as a result of sensory feedback at each event. The sensory feedback provided at each event changes the relative phases.

We use the timing immediately before event T2 for the Poincaré section and find the fixed point for the relative phases $\left({\hat{\psi }}_{1}^{\text{T2}},\;{\hat{\psi }}_{2}^{\text{T2}}\right)$ to produce the periodic solution; here, we denote the value immediately before event T*i* by ${\left(\right)}^{\text{T}i}$ and the value of the periodic solution immediately before event T*i* by ${\hat{\left(\right)}}^{\text{T}i}$. Phases (*ϕ*_1_, *ϕ*_2_, *ϕ*_3_) evolve over time, and the relative phases (*ψ*_1_, *ψ*_2_) evolve as events occur. Fig 14 shows how the phases (*ϕ*_1_, *ϕ*_2_, *ϕ*_3_) and the relative phases (*ψ*_1_, *ψ*_2_) at each event evolve as a result of the sensory feedback, where event T2’ indicates event T2 after half a gait cycle, and the value next to “Sensory feedback” indicates the amount of change in the oscillator phase caused by phase resetting (21). Immediately before event T2 (the Poincaré section), $\phi_{2}^{\text{T2}}$ is equal to $\phi_{2}^{\text{td}}$, and the relative phases are represented by $\left(\psi_{1}^{\text{T2}},\;\psi_{2}^{\text{T2}}\right)$, as per Fig 14. Immediately after event T2, the phase *ϕ*_2_ is changed to $\pi +\phi_{2}^{\text{td}}/2$ by the sensory feedback. The relative phases immediately after event T2 are also changed as shown in Fig 14 because of the sensory feedback. Next, event T3 occurs. Immediately before event T3, $\phi_{3}^{\text{T3}}$ is equal to $\phi_{3}^{\text{td}}$. Because no leg touchdown event occurs between events T2 and T3, the relative phases immediately before event T3 are the same as those immediately after event T2, which is indicated by the equals sign in Fig 14. While we omit further explanation of Fig 14 here, we can see that the state variables $\left(\phi_{1}^{\text{T}i},\;\phi_{2}^{\text{T}i},\;\phi_{3}^{\text{T}i}\right)$ immediately before each event T*i* are represented by the relative phases on the Poincaré section $\left(\psi_{1}^{\text{T2}},\;\psi_{2}^{\text{T2}}\right)$ and $\left(\phi_{1}^{\text{td}},\;\phi_{2}^{\text{td}},\;\phi_{3}^{\text{td}}\right)$. To find the periodic solution, we then solve for ${\hat{\phi }}_{2}^{\text{td}}\left(={\hat{\phi }}_{1}^{\text{td}}={\hat{\phi }}_{3}^{\text{td}}\right)$, ${\hat{\psi }}_{1}^{\text{T2}}$, and ${\hat{\psi }}_{2}^{\text{T2}}$, which can be determined from the phase relationship $\Delta l_{1}^{*}=0$, $\Delta l_{2}^{*}=0$, and $\Delta l_{3}^{*}=0$ immediately before events T1, T2, and T3, respectively. As a result, ${\hat{\psi }}_{1}^{\text{T2}}$ and ${\hat{\psi }}_{2}^{\text{T2}}$ are given by $\psi_{1}^{\text{Dw}}$ and $\psi_{2}^{\text{Dw}}$, respectively (see (3)). A detailed explanation of this derivation is presented in S3 Appendix.

We investigate the stability of this direct wave gait by analyzing how the small perturbations Δ*ψ*_1_ and Δ*ψ*_2_ for the relative phases ${\hat{\psi }}_{1}^{\text{T2}}$ and ${\hat{\psi }}_{2}^{\text{T2}}$ immediately before event T2 evolve after a single gait cycle, where we assume that these perturbations do not change the sets of stance legs *S*_T1_, *S*_T2_, and *S*_T3_. We obtain the perturbations after they have evolved over one gait cycle $\left(\Delta \psi_{1}^{\prime },\;\Delta \psi_{2}^{\prime }\right)$ from the amount of phase resetting that occurs at each event using

$$\begin{array}{c} \left[\begin{array}{c} \Delta \psi_{1}^{\prime }\\ \Delta \psi_{2}^{\prime } \end{array}\right]={\left[\begin{array}{cc} \sqrt{\lambda_{1}^{\text{Dw}}} & 0\\ \sqrt{\lambda_{2}^{\text{Dw}}}\left(1-\sqrt{\lambda_{1}^{\text{Dw}}}\right) & \sqrt{\lambda_{2}^{\text{Dw}}} \end{array}\right]}^{2}\left[\begin{array}{c} \Delta \psi_{1}\\ \Delta \psi_{2} \end{array}\right], \end{array}$$ (23)

where

$$\begin{array}{ccc} \lambda_{1}^{\text{Dw}} & = & {\left(\frac{5}{6}-\frac{4}{45\beta }\frac{s^{*}}{a^{*}}\right)}^{2},\\ \lambda_{2}^{\text{Dw}} & = & {\left(\frac{13}{18}+\frac{4}{81\beta }\frac{s^{*}}{a^{*}}\right)}^{2}. \end{array}$$ (24)

A detailed explanation of how we obtain this solution is presented in S4 Appendix. $\lambda_{1}^{\text{Dw}}$ and $\lambda_{2}^{\text{Dw}}$ correspond to the eigenvalues of the evolution matrix of the perturbations. Because s* is small ($O({\left(K^{*}\right)}^{-\frac{2}{3}})$), $\lambda_{1}^{\text{Dw}}\left(=\lambda^{\text{Dw}}\right)$ is the maximum eigenvalue and thus determines the stability of this gait.

**Retrograde wave gait** Next, we derive the periodic solution for the retrograde wave gait and investigate its stability by considering the conditions of symmetry between the direct and retrograde wave gaits. We denote the flow of the oscillator phases by the stride parameter s* and the initial values $\phi_{1}^{\text{T2}},\;\phi_{2}^{\text{T2}}$, and $\phi_{3}^{\text{T2}}$ immediately before event T2 by $\Phi_{s^{*}}\left(t;\;\phi_{1}^{\text{T2}},\;\phi_{2}^{\text{T2}},\;\phi_{3}^{\text{T2}}\right)$. Because the model movements for the direct wave gait with s* > 0 and for the retrograde wave gait with −s* < 0 are identical except for the walking direction, we can then write

$$\begin{array}{c} \Phi_{s^{*}}\left(t;\;\phi_{1}^{\text{T2}},\;\phi_{2}^{\text{T2}},\;\phi_{3}^{\text{T2}}\right)=\Phi_{-s^{*}}\left(t;\;\phi_{3}^{\text{T2}},\;\phi_{2}^{\text{T2}},\;\phi_{1}^{\text{T2}}\right) \end{array}$$ (25)

Based on this symmetry condition, ${\hat{\psi }}_{1}^{\text{T2}}$ and ${\hat{\psi }}_{2}^{\text{T2}}$ for the retrograde wave gait are given by $\psi_{1}^{\text{Rw}}$ and $\psi_{2}^{\text{Rw}}$, respectively (see (4)). Additionally, the eigenvalues of the evolution matrix $\left(\lambda_{1}^{\text{Rw}},\;\lambda_{2}^{\text{Rw}}\right)$ become

$$\begin{array}{ccc} \lambda_{1}^{\text{Rw}} & = & {\left(\frac{5}{6}+\frac{4}{45\beta }\frac{s^{*}}{a^{*}}\right)}^{2},\\ \lambda_{2}^{\text{Rw}} & = & {\left(\frac{13}{18}-\frac{4}{81\beta }\frac{s^{*}}{a^{*}}\right)}^{2}. \end{array}$$ (26)

The maximum eigenvalue $\lambda_{1}^{\text{Rw}}\left(=\lambda^{\text{Rw}}\right)$ thus determines the stability of this gait.

**Stability mechanism** In this section, the mechanism by which the perturbations evolve in this stability analysis is explained briefly by focusing on a specific leg. The perturbations in the relative phases change the body inclination through the elasticity of the leg and thus change the timing of the leg touchdown, which induces changes in the relative phases through phase resetting, as shown in S3 and S4 Appendixes. As a result, the perturbations change after the leg touchdown event. This process reduces the perturbations after a single gait cycle through six leg touchdown events, as shown in (24) and (26).

In addition, because the legs propel the body (s* ≠ 0), the relative foot positions between the legs at the leg touchdown point are different for the two gaits (direct and retrograde wave gaits), as characterized by (25). Therefore, the body inclination angles induced by the perturbation are different for the two gaits. This changes the phase resetting intensity, and the stability then differs between the two gaits, as characterized by the length s*/a* in (24) and (26).

## Supporting information

S1 Movie Direct wave gait in the robot experiments. This movie shows the direct wave gaits of the robot at a duty factor of *β* = 0.6. The swing movement propagates from back to front. Additionally, this is the metachronal gait because all four legs are almost always in contact with the ground.
(MP4)

S2 Movie Retrograde wave gait in the robot experiments. This movie shows the retrograde wave gaits of the robot at a duty factor of *β* = 0.6. While all four legs are almost always in contact with the ground, the swing movement propagates from front to back.
(MP4)

S1 Appendix Effect of the phase interaction between left and right on phase resetting. This appendix explains how assumptions A6 and A7 reduce the original phase dynamics of (6) to the reduced phase dynamics of (21).
(PDF)

S2 Appendix Set of stance legs immediately before each event for the direct wave gait. This appendix explains how the set of stance legs immediately before each event is determined for the direct wave gait.
(PDF)

S3 Appendix Derivation of periodic solution for the direct wave gait. This appendix explains how the periodic solution for the direct wave gait in (3) is obtained.
(PDF)

S4 Appendix Stability analysis of the direct wave gait. This appendix explains how the evolution matrix of the perturbations in (23) is derived.
(PDF)
