# Supplementary material for: Simple analytical model reveals the functional role of embodied sensorimotor interaction in hexapod gaits
Source: PLoS One. 2018 Feb 28;13(2):e0192469. doi: 10.1371/journal.pone.0192469 (PMC5831041; doi:10.1371/journal.pone.0192469)
Supplement: S1 Appendix — This appendix explains how assumptions A6 and A7 reduce the original phase dynamics of (6) to the reduced phase dynamics of (21). (PDF) [file pone.0192469.s003.pdf]

S1 Appendix Effect of the phase interaction between left and right on the phase resetting

In this appendix, we show how assumptions A6 and 7 influence sensory feedback by phase resetting in the phase dynamics (6).

From assumption A7 ($\tau = 0$), the phase dynamics for the left and right legs becomes

$$\begin{aligned} \dot{\phi}_r &= \omega - k_{\mathrm{c}} \sin(\phi_r - \phi_l - \pi) + k_{\mathrm{f}}^r, \\ \dot{\phi}_l &= \omega - k_{\mathrm{c}} \sin(\phi_l - \phi_r - \pi) + k_{\mathrm{f}}^l, \end{aligned} \tag{S1.1}$$

where $(r, l) = \{(1, 4), (2, 5), (3, 6)\}$. When phase resetting does not occur ($k_{\mathrm{f}}^r = k_{\mathrm{f}}^l = 0$), these equations yield

$$\frac{d}{dt}(\phi_r - \phi_l) = -2k_{\mathrm{c}} \sin(\phi_r - \phi_l - \pi). \tag{S1.2}$$

Then, the relative phase $\phi_r - \phi_l$ is given by the first order approximation about $\phi_r - \phi_l = \pi$ as follows:

$$\phi_r(t) - \phi_l(t) = (\phi_r(t_{\mathrm{o}}) - \phi_l(t_{\mathrm{o}}) - \pi)\, e^{-2k_{\mathrm{c}}(t - t_{\mathrm{o}})} + \pi. \tag{S1.3}$$

We suppose that phase resetting occurs for ϕ_r at $t = t_{\mathrm{o}}$ and that $\phi_r(t_{\mathrm{o}}^-) = 2\pi - \Delta$ and $\phi_l(t_{\mathrm{o}}^-) = \pi - \Delta$ from the assumption A6, where $t = t_{\mathrm{o}}^-$ is the time immediately before $t = t_{\mathrm{o}}$ and $\Delta \ll 1$. Then, phase resetting yields $\phi_r(t_{\mathrm{o}}^+) = 0$ and $\phi_l(t_{\mathrm{o}}^+) = \pi - \Delta$, where $t = t_{\mathrm{o}}^+$ is the time immediately after $t = t_{\mathrm{o}}$ and Δ corresponds to the phase reset value. Because $\phi_r(t_{\mathrm{o}}^+) - \phi_l(t_{\mathrm{o}}^+) = \Delta + \pi \in [0, 2\pi)$ and $\Delta \ll 1$, the relative phase $\phi_r(t) - \phi_l(t)$ after the phase resetting is given using (S1.3) by

$$\phi_r(t) - \phi_l(t) = \Delta e^{-2k_{\mathrm{c}}(t - t_{\mathrm{o}})} + \pi, \quad t > t_{\mathrm{o}}. \tag{S1.4}$$

Then, substituting (S1.4) for (S1.1), the phase dynamics of ϕ_r are given by

$$\dot{\phi}_r = \omega - k_{\mathrm{c}} \sin(\Delta e^{-2k_{\mathrm{c}}(t - t_{\mathrm{o}})}) + k_{\mathrm{f}}^r, \quad t > t_{\mathrm{o}}. \tag{S1.5}$$

Until the next phase resetting occurs, ϕ_r can be written as a first order approximation of Δ as follows:

$$\phi_r = \omega(t - t_{\mathrm{o}}) + \frac{1}{2}\Delta e^{-2k_{\mathrm{c}}(t - t_{\mathrm{o}})} - \frac{1}{2}\Delta, \quad t > t_{\mathrm{o}}. \tag{S1.6}$$

After a sufficient duration ($\gg 1/k_{\mathrm{c}}$) after phase resetting, ϕ_r becomes $\omega(t - t_{\mathrm{o}}) - \frac{1}{2}\Delta$. This means that assumptions A6 and A7 reduce the phase reset value from Δ to $\frac{1}{2}\Delta$. Therefore, we used the coefficient 1/2 for the phase resetting term k_{f} in (21).
