# Supplementary material for: Simple analytical model reveals the functional role of embodied sensorimotor interaction in hexapod gaits
Source: PLoS One. 2018 Feb 28;13(2):e0192469. doi: 10.1371/journal.pone.0192469 (PMC5831041; doi:10.1371/journal.pone.0192469)
Supplement: S2 Appendix — This appendix explains how the set of stance legs immediately before each event is determined for the direct wave gait. (PDF) [file pone.0192469.s004.pdf]

S2 Appendix Set of stance legs immediately before each event for the direct wave gait

In this appendix, we explain how the set of stance legs immediately before each event is determined for the direct wave gait. Suppose $\psi_1 = \psi_2 = 2(1-\beta)\pi$, then the oscillator phases are written using β as shown in Fig. S2.1, where $\phi_i = 0$ at touchdown. In the range $1/2 < \beta < 2/3$, we assume that the leg is contact with the ground for $0 < \phi_i \leq 2\beta\pi$, which includes the liftoff event, and in the air for $2\beta\pi < \phi_i \leq 2\pi$, which includes the touchdown event. These assumptions give the sets of stance legs as in (22).

Because the analytical solution is $\psi_1 = \psi_2 = 2(1-\beta)\pi + O((K^*)^{-1})$ as in (3), the estimated set of stance legs may not be correct at $\phi_i \sim 2\beta\pi$ or 2π because of $O((K^*)^{-1})$, especially for $\beta \sim 1/2$ and $2/3$. However, we ignore these possibilities because we focus on the global behavior of the solution in $1/2 < \beta < 2/3$.

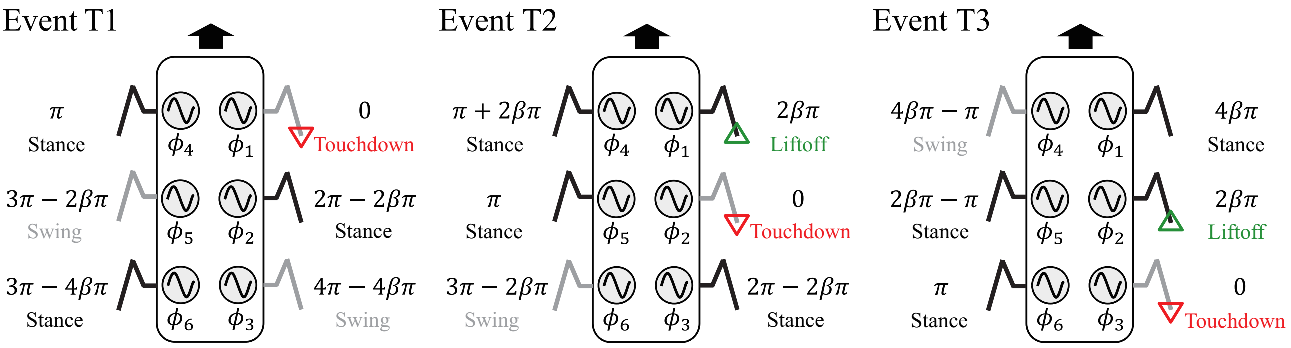

Figure S2.1: Oscillator phase values at events T1, T2, and T3
