# Supplementary material for: Simple analytical model reveals the functional role of embodied sensorimotor interaction in hexapod gaits
Source: PLoS One. 2018 Feb 28;13(2):e0192469. doi: 10.1371/journal.pone.0192469 (PMC5831041; doi:10.1371/journal.pone.0192469)
Supplement: S3 Appendix — This appendix explains how the periodic solution for the direct wave gait in (3) is obtained. (PDF) [file pone.0192469.s005.pdf]

S3 Appendix Derivation of periodic solution of the direct wave gait

In this appendix, we explain how we obtained the periodic solution of the direct wave gait in the Results section by solving $\hat{\phi}_2^{\rm td}$, $\hat{\psi}_1^{\rm T2}$, and $\hat{\psi}_2^{\rm T2}$ immediately before event T2. More specifically, we represent the oscillator phases immediately before each event by these variables, and then derive the condition $\Delta l_i^* = 0$ of each event. After that, we solve these variables.

1) The phases and condition $\Delta l_1^* = 0$ immediately before event T1

The phases immediately before event T1 are given as follows using the condition for periodic solution $\hat{\phi}_1^{\rm td} = \hat{\phi}_2^{\rm td} = \hat{\phi}_3^{\rm td}$ and the phase relationship in Fig. 14:

$$
\begin{aligned}
\phi_1^{\rm T1} &= \hat{\phi}_2^{\rm td}, \\
\phi_2^{\rm T1} &= \frac{1}{2}\hat{\phi}_2^{\rm td} + \hat{\psi}_1^{\rm T2} - \pi, \\
\phi_3^{\rm T1} &= \frac{1}{2}\hat{\phi}_2^{\rm td} + \hat{\psi}_1^{\rm T2} + \hat{\psi}_2^{\rm T2} - \pi.
\end{aligned}
\tag{S3.1}
$$

The vertical distance from the root of Leg 1 to the ground $L_1^{\rm r*}$ immediately before event T1 is obtained from the equilibrium conditions in (18), (19), and (20), and $S_{\rm T1}$ in (22) as follows:

$$
L_1^{\rm r*} = 1 - \frac{1}{2K^*}. \tag{S3.2}
$$

When Leg 1 touches the ground, the vertical distance from the root of Leg 1 to the ground $L_1^{\rm r*}$ is identical to the nominal length of Leg 1 determined by the phase $\phi_1^{\rm T1}$ ($\Delta l_1^* = 0$). The nominal length of Leg 1 is given substituting $\phi_i = \phi_1^{\rm T1}$ into normalized (11) as

$$
L_1^{\rm r*} = 1 - d^* \sin\frac{\phi_1^{\rm T1} - 2\beta\pi}{2(1-\beta)}. \tag{S3.3}
$$

Then, using the first order approximation about $\phi_1^{\rm T1} = 2\pi$ yields the following phase relationship:

$$
L_1^{\rm r*} = 1 - \frac{(2\pi - \phi_1^{\rm T1})d^*}{2(1-\beta)} + O((K^*)^{-2}), \tag{S3.4}
$$

where $2\pi - \phi_1^{\rm T1} \sim O((K^*)^{-1})$.

2) The phases and condition $\Delta l_2^* = 0$ immediately before event T2

The phases immediately before event T2 are given as follows using the condition for periodic solution and the phase relationship in Fig. 14:

$$
\begin{aligned}
\phi_1^{\rm T2} &= \hat{\phi}_2^{\rm td} - \hat{\psi}_1^{\rm T2}, \\
\phi_2^{\rm T2} &= \hat{\phi}_2^{\rm td}, \\
\phi_3^{\rm T2} &= \hat{\phi}_2^{\rm td} + \hat{\psi}_2^{\rm T2} - 2\pi.
\end{aligned}
\tag{S3.5}
$$

The vertical distance from the root of Leg 2 to the ground $L_2^{\rm r*}$ immediately before event T2 is given by a function of $(\phi_1^{\rm T2}, \phi_2^{\rm T2}, \phi_3^{\rm T2})$ from Δx_i^* in (12), the equilibrium conditions in (18), (19), and (20), and

S_{T2} in (22) as follows:

$$L_2^{\mathrm{r}*} = 1 + \left(a_{\mathrm{T2}} - \frac{1}{2}\right)\frac{(\phi_1^{\mathrm{T2}} - 2\beta\pi)d^*}{2(1-\beta)} + \left(b_{\mathrm{T2}} - \frac{1}{4}\right)\frac{1}{K^*} + O((K^*)^{-2}), \tag{S3.6}$$

where

$$\begin{aligned} a_{\mathrm{T2}} &= \frac{1}{5a^*}\left\{\Delta x_1^*(\phi_1^{\mathrm{T2}}) - 2\Delta x_2^*(\phi_2^{\mathrm{T2}}) + \Delta x_3^*(\phi_3^{\mathrm{T2}})\right\}, \\ b_{\mathrm{T2}} &= -\frac{1}{20a^*}\left\{\Delta x_1^*(\phi_1^{\mathrm{T2}}) - 2\Delta x_2^*(\phi_2^{\mathrm{T2}}) + \Delta x_3^*(\phi_3^{\mathrm{T2}})\right\}, \end{aligned}$$

$2\beta\pi \le \{\phi_1^{\mathrm{T2}}, \phi_2^{\mathrm{T2}}\} < 2\pi$, $0 \le \phi_3^{\mathrm{T2}} < 2\beta\pi$, and $\phi_1^{\mathrm{T2}} - 2\beta\pi \sim O((K^*)^{-1})$.

When Leg 2 touches the ground, the vertical distance from the root of Leg 2 to the ground ${L_2^{\mathrm{r}}}^*$ is identical to the nominal length of Leg 2 determined by the phase ϕ_2^{td} ($\Delta l_2^* = 0$). The nominal length of Leg 2 is given substituting $\phi_i = \phi_2^{\mathrm{T2}}$ into normalized (11) as

$$L_2^{\mathrm{r}*} = 1 - d^* \sin\frac{\phi_2^{\mathrm{T2}} - 2\beta\pi}{2(1-\beta)}. \tag{S3.7}$$

Then, using the first order approximation about $\phi_2^{\mathrm{T2}} = 2\pi$ yields the following phase relationship:

$$L_2^{\mathrm{r}*} = 1 - \frac{(2\pi - \phi_2^{\mathrm{T2}})d^*}{2(1-\beta)} + O((K^*)^{-2}), \tag{S3.8}$$

where $2\pi - \phi_2^{\mathrm{T2}} \sim O((K^*)^{-1})$.

3) The phases and condition $\Delta l_3^* = 0$ immediately before event T3

The phases immediately before event T3 are given as follows using the condition for periodic solution and the phase relationship in Fig. 14:

$$\begin{aligned} \phi_1^{\mathrm{T3}} &= \hat{\phi}_2^{\mathrm{td}} - \hat{\psi}_1^{\mathrm{T2}} - \hat{\psi}_2^{\mathrm{T2}}, \\ \phi_2^{\mathrm{T3}} &= \frac{1}{2}\hat{\phi}_2^{\mathrm{td}} - \hat{\psi}_2^{\mathrm{T2}} + \pi, \\ \phi_3^{\mathrm{T3}} &= \hat{\phi}_2^{\mathrm{td}}. \end{aligned} \tag{S3.9}$$

The vertical distance from the root of Leg 3 to the ground $L_3^{\mathrm{r}*}$ immediately before event T3 is obtained as a function of $(\phi_1^{\mathrm{T3}}, \phi_2^{\mathrm{T3}}, \phi_3^{\mathrm{T3}})$ from Δx_i^* in (12), the equilibrium conditions in (18), (19), and (20), and S_{T3} in (22) as follows:

$$L_3^{\mathrm{r}*} = 1 + \left(a_{\mathrm{T3}} - \frac{5}{4}\right)\frac{(\phi_2^{\mathrm{T3}} - 2\beta\pi)d^*}{2(1-\beta)} + \left(b_{\mathrm{T3}} - \frac{1}{4}\right)\frac{1}{K^*} + O((K^*)^{-2}), \tag{S3.10}$$

where

$$\begin{aligned} a_{\mathrm{T3}} &= \frac{1}{4a^*}\left\{-\Delta x_1^*(\phi_1^{\mathrm{T3}}) - \Delta x_2^*(\phi_2^{\mathrm{T3}}) + 2\Delta x_3^*(\phi_3^{\mathrm{T3}}) + 3\Delta x_5^*(\phi_2^{\mathrm{T3}} + \pi) - 3\Delta x_6^*(\phi_3^{\mathrm{T3}} + \pi)\right\}, \\ b_{\mathrm{T3}} &= -\frac{3}{8a^*}\left\{\Delta x_1^*(\phi_1^{\mathrm{T3}}) + \Delta x_2^*(\phi_2^{\mathrm{T3}}) + \Delta x_5^*(\phi_2^{\mathrm{T3}} + \pi) + \Delta x_6^*(\phi_3^{\mathrm{T3}} + \pi)\right\}. \end{aligned}$$

$2\beta\pi \le \{\phi_2^{\mathrm{T3}}, \phi_3^{\mathrm{T3}}\} < 2\pi$, $0 \le \phi_1^{\mathrm{T3}} < 2\beta\pi$, and $\phi_2^{\mathrm{T3}} - 2\beta\pi \sim O((K^*)^{-1})$.

When Leg 3 touches the ground, the vertical distance from the root of Leg 3 to the ground ${L_3^{\mathrm{r}}}^*$ is identical to the nominal length of Leg 3 determined by the phase ϕ_3^{T3} ($\Delta l_3^* = 0$). The nominal length of Leg 3 is given substituting $\phi_i = \phi_3^{\mathrm{T3}}$ into normalized (11) as

$$L_3^{\mathrm{r}*} = 1 - d^* \sin \frac{\phi_3^{\mathrm{T3}} - 2\beta\pi}{2(1-\beta)}. \tag{S3.11}$$

Then, using the first order approximation about $\phi_3^{\mathrm{T3}} = 2\pi$ yields the following phase relationship:

$$L_3^{\mathrm{r}*} = 1 - \frac{(2\pi - \phi_3^{\mathrm{T3}})d^*}{2(1-\beta)} + O((K^*)^{-2}), \tag{S3.12}$$

where $2\pi - \phi_3^{\mathrm{T3}} \sim O((K^*)^{-1})$.

4) Derivation of the periodic solution

$\hat{\phi}_2^{\mathrm{td}}$ is given from (S3.2) and (S3.4) by

$$\hat{\phi}_2^{\mathrm{td}} = 2\pi - (1-\beta)\frac{1}{d^* K^*} + O((K^*)^{-2}). \tag{S3.13}$$

$\hat{\psi}_1^{\mathrm{T2}}$ and $\hat{\psi}_2^{\mathrm{T2}}$ are obtained from (S3.6), (S3.8), (S3.10), and (S3.12) as (3) ($\hat{\psi}_1^{\mathrm{T2}} = \psi_1^{\mathrm{Dw}}$ and $\hat{\psi}_2^{\mathrm{T2}} = \psi_2^{\mathrm{Dw}}$).
