# Supplementary material for: Simple analytical model reveals the functional role of embodied sensorimotor interaction in hexapod gaits
Source: PLoS One. 2018 Feb 28;13(2):e0192469. doi: 10.1371/journal.pone.0192469 (PMC5831041; doi:10.1371/journal.pone.0192469)
Supplement: S4 Appendix — This appendix explains how the evolution matrix of the perturbations in (23) is derived. (PDF) [file pone.0192469.s006.pdf]

S4 Appendix Stability analysis of the direct wave gait

In this appendix, we derive the evolution matrix of perturbations (23) in the Results section. More specifically, we add small perturbations ($\Delta\psi_1$, $\Delta\psi_2$) immediately before event T2 and analyze how the perturbations evolve after half a gait cycle (sequential events: T2, T3, and T1); i.e., we derive the amount of sensory feedback of three events. Suppose that the perturbations are small to be $O((K^*)^{-1})$, which do not change the sets of stance legs S_{T1}, S_{T2}, and S_{T3}.

1) Amount of sensory feedback of event T2

When we put the perturbations $\Delta\psi_1$ and $\Delta\psi_2$ immediately before event T2, the phases immediately before event T2 are given by

$$\begin{aligned} \phi_1^{\mathrm{T2}} &= \phi_2^{\mathrm{td}} - \psi_1^{\mathrm{T2}}, \\ \phi_2^{\mathrm{T2}} &= \phi_2^{\mathrm{td}}, \\ \phi_3^{\mathrm{T2}} &= \phi_2^{\mathrm{td}} + \psi_2^{\mathrm{T2}} - 2\pi, \end{aligned} \tag{S4.1}$$

where

$$\begin{aligned} \psi_1^{\mathrm{T2}} &= \hat{\psi}_1^{\mathrm{T2}} + \Delta\psi_1, \\ \psi_2^{\mathrm{T2}} &= \hat{\psi}_2^{\mathrm{T2}} + \Delta\psi_2. \end{aligned}$$

We obtain ϕ_2^{td} by substituting (S4.1) into (S3.6) and (S3.8) as

$$\begin{aligned} \phi_2^{\mathrm{td}} &= 2\pi - (1-\beta)\frac{1}{d^*K^*} + 2(1-c_1)\Delta\psi_1, \\ c_1 &= \frac{5}{6} - \frac{4}{45\beta}\frac{s^*}{a^*}. \end{aligned} \tag{S4.2}$$

From Fig. 14, the amount of sensory feedback of event T2 is given by $(1-\beta)/(2d^*K^*) - (1-c_1)\Delta\psi_1$.

2) Amount of sensory feedback of event T3

Using the amount of sensory feedback of event T2, the phases immediately before event T3 are given by (Fig. 14)

$$\begin{aligned} \phi_1^{\mathrm{T3}} &= \phi_3^{\mathrm{td}} - \psi_1^{\mathrm{T3}} - \psi_2^{\mathrm{T3}}, \\ \phi_2^{\mathrm{T3}} &= \phi_3^{\mathrm{td}} - \psi_2^{\mathrm{T3}}, \\ \phi_3^{\mathrm{T3}} &= \phi_3^{\mathrm{td}}, \end{aligned} \tag{S4.3}$$

where

$$\begin{aligned} \psi_1^{\mathrm{T3}} &= \hat{\psi}_1^{\mathrm{T2}} + \frac{1}{2}(1-\beta)\frac{1}{d^*K^*} + c_1\Delta\psi_1, \\ \psi_2^{\mathrm{T3}} &= \hat{\psi}_2^{\mathrm{T2}} - \frac{1}{2}(1-\beta)\frac{1}{d^*K^*} + (1-c_1)\Delta\psi_1 + \Delta\psi_2. \end{aligned}$$

We obtain ϕ_3^{td} by substituting (S4.3) into (S3.10) and (S3.12) as

$$\begin{aligned} \phi_3^{\mathrm{td}} &= 2\pi - (1-\beta)\frac{1}{d^* K^*} + 2(1-c_2)(1-c_1)\Delta\psi_1 + 2(1-c_2)\Delta\psi_2, \\ c_2 &= \frac{13}{18} + \frac{4}{81\beta}\frac{s^*}{a^*}. \end{aligned} \tag{S4.4}$$

From Fig. 14, the amount of sensory feedback of event T3 is given by $(1-\beta)/(2d^* K^*) - (1-c_2)(1-c_1)\Delta\psi_1 - (1-c_2)\Delta\psi_2$.

3) Amount of sensory feedback of event T1

Using the amount of sensory feedback of event T3, the phases immediately before event T1 are given by (Fig. 14)

$$\begin{aligned} \phi_1^{\mathrm{T1}} &= \phi_1^{\mathrm{td}}, \\ \phi_2^{\mathrm{T1}} &= \phi_1^{\mathrm{td}} + \psi_1^{\mathrm{T1}} - 2\pi, \\ \phi_3^{\mathrm{T1}} &= \phi_1^{\mathrm{td}} + \psi_1^{\mathrm{T1}} + \psi_2^{\mathrm{T1}} - 2\pi, \end{aligned} \tag{S4.5}$$

where

$$\begin{aligned} \psi_1^{\mathrm{T1}} &= \hat{\psi}_1^{\mathrm{T2}} + \frac{1}{2}(1-\beta)\frac{1}{d^* K^*} + c_1\Delta\psi_1, \\ \psi_2^{\mathrm{T1}} &= \hat{\psi}_2^{\mathrm{T2}} + c_2(1-c_1)\Delta\psi_1 + c_2\Delta\psi_2. \end{aligned}$$

We obtain ϕ_1^{td} from (S3.2) and (S3.4) as

$$\phi_1^{\mathrm{td}} = 2\pi - (1-\beta)\frac{1}{d^* K^*}. \tag{S4.6}$$

From Fig. 14, the amount of sensory feedback of event T1 is given by $(1-\beta)/(2d^* K^*)$.

4) Relative phases immediately before event T2 after one gait cycle

Using the amount of sensory feedback of event T1, relative phases $\psi_1^{\mathrm{T2}\prime}$ and $\psi_2^{\mathrm{T2}\prime}$ immediately before event T2 after half a gait cycle are given by (Fig. 14)

$$\begin{aligned} \psi_1^{\mathrm{T2}\prime} &= \hat{\psi}_1^{\mathrm{T2}} + c_1\Delta\psi_1, \\ \psi_2^{\mathrm{T2}\prime} &= \hat{\psi}_2^{\mathrm{T2}} + c_2(1-c_1)\Delta\psi_1 + c_2\Delta\psi_2. \end{aligned} \tag{S4.7}$$

The same procedure is applicable to the latter half of the gait cycle because the order of the touchdown events is identical, and we obtain the evolution matrix of the perturbations as (23).
